# Associations of DNA Base Excision Repair and Antioxidant Enzyme Genetic Risk Scores with Biomarker of Systemic Inflammation

**DOI:** 10.3389/fragi.2022.897907

**Published:** 2022-05-04

**Authors:** Ziling Mao, Abigail L. H. Gray, Myron D. Gross, Bharat Thyagarajan, Roberd M. Bostick

**Affiliations:** ^1^ Department of Epidemiology, Rollins School of Public Health, Emory University, Atlanta, GA, United States; ^2^ Department of Laboratory Medicine and Pathology, University of Minnesota, Minnesota, MN, United States; ^3^ Winship Cancer Institute, Emory University, Atlanta, GA, United States

**Keywords:** inflammation biomarker, high sensitivity C-reactive protein, antioxidant enzymes genotypes, DNA base excision repair genotypes, genetic risk scores, inflammation scores

## Abstract

**Background:** Inflammation is implicated in the etiology of various aging-related diseases. Numerous dietary and lifestyle factors contribute to chronic systemic inflammation; genetic variation may too. However, despite biological plausibility, little is known about associations of antioxidant enzyme (AE) and DNA base excision repair (BER) genotypes with human systemic inflammation.

**Methods:** We genotyped 22 single nucleotide polymorphisms (SNPs) in 3 AE genes, and 79 SNPs in 14 BER genes to develop inflammation-specific AE and BER genetic risk scores (GRS) in two pooled cross-sectional studies (*n* = 333) of 30–74-year-old White adults without inflammatory bowel disease, familial adenomatous polyposis, or a history of cancer or colorectal adenoma. Of the genotypes, based on their associations with a biomarker of systemic inflammation, circulating high sensitivity C-reactive protein (hsCRP) concentrations, we selected 2 SNPs of 2 genes (*CAT* and *MnSoD*) for an AE GRS, and 7 SNPs of 5 genes (*MUTYH*, *SMUG1*, *TDG*, *UNG*, and *XRCC1*) for a BER GRS. A higher GRS indicates a higher balance of variant alleles directly associated with hsCRP relative to variant alleles inversely associated with hsCRP. We also calculated previously-reported, validated, questionnaire-based dietary (DIS) and lifestyle (LIS) inflammation scores. We used multivariable general linear regression to compare mean hsCRP concentrations across AE and BER GRS categories, individually and jointly with the DIS and LIS.

**Results:** The mean hsCRP concentrations among those in the highest relative to the lowest AE and BER GRS categories were, proportionately, 13.9% (*p* = 0.30) and 57.4% (*p* = 0.009) higher. Neither GRS clearly appeared to modify the associations of the DIS or LIS with hsCRP.

**Conclusion:** Our findings suggest that genotypes of DNA BER genes collectively may be associated with systemic inflammation in humans.

## 1 Introduction

Substantial epidemiological evidence suggests that chronic inflammation is associated with various aging phenotypes, such as metabolic homeostasis, immune senescence, and changes in body composition (Franceschi and Campisi, 2014). Inflammation is also implicated in the etiology of multiple aging-related diseases (Sanada et al., 2018), including cardiovascular disease and cancer (Gan et al., 2004; Pawelec et al., 2014; Singh et al., 2019). Dietary and lifestyle exposures likely contribute to higher chronic systemic inflammation (Cavicchia et al., 2009; Barbaresko et al., 2013; Kantor et al., 2013), and genetic variation may too (Shen and Ordovas, 2009). Since reactive oxygen species (ROS) play an important role in inflammation and the pathogenesis of many inflammatory diseases (Mittal et al., 2014), and antioxidant enzymes (AE) and DNA base excision repair (BER) mechanisms contribute to protecting cells from ROS-induced deleterious effects (Sun, 1990; Seeberg et al., 1995; Mates, 2000), polymorphisms in AE and BER genes may be especially important in regulating systemic inflammation.

Substantial basic science and human study evidence supports that multiple dietary constituents and lifestyle factors contribute to systemic inflammation, and recent epidemiologic evidence consistently, strongly supports that dietary and lifestyle factors collectively are particularly strongly associated with circulating concentrations of inflammation biomarkers and chronic diseases for which inflammation is considered an important risk factor. Circulating concentrations of high sensitivity C-reactive protein (hsCRP), an acute phase protein produced in the liver and released into the circulation, has been used extensively clinically and in epidemiologic studies (Byrd et al., 2019) as a biomarker of inflammation. Although, for some applications, it has some limitations (e.g., increases in response to infection and other inflammatory medical conditions (Sproston and Ashworth, 2018)), it is reliably measured (Ridker, 2004), is the most widely used biomarker of inflammation in epidemiologic studies, and is highly correlated with other biomarkers of inflammation (Byrd et al., 2019). We recently reported the development and validation of novel, questionnaire-based, dietary and lifestyle inflammation scores, the DIS and LIS, respectively (Byrd et al., 2019). The components of the predominantly whole foods-based, 19-component DIS and the 4-component LIS [comprising physical activity, body mass index (BMI), alcohol intake, and smoking status] are weighted according to the strengths of their associations with a panel of inflammation biomarkers (including hsCRP) in a diverse population of Black and White male and female adults from the 48 contiguous United States. The DIS was more strongly associated with inflammation biomarkers than were other reported dietary inflammation indices in three populations (one of which included participants in the present study), and the strongest associations were among those who were jointly in the highest DIS and LIS categories (Byrd et al., 2019).

Just as individual dietary and lifestyle factors, for the most part, tend to be modestly and inconsistently associated with inflammation, but collectively are strongly and consistently associated with it (Byrd et al., 2019), it is unlikely that single, relatively common AE or BER polymorphisms would be strongly associated with systemic inflammation, but collectively they might. Genetic risk scores (GRS) that combine multiple variants in multiple genes were previously reported and found to be associated with chronic disease outcomes (Wang et al., 2017; Chalmer et al., 2018; Korologou-Linden et al., 2019a; Korologou-Linden et al., 2019b; Leonenko et al., 2019; Mavaddat et al., 2019; Watt et al., 2019; Zheutlin et al., 2019; Li et al., 2020; Mosley et al., 2020; Sipeky et al., 2020). Associations of AE and BER GRS with colorectal adenoma were previously reported (Wang et al., 2017); however, there are no reported associations of these or other GRS, alone or in interaction with dietary and lifestyle exposures, with inflammation biomarkers.

Accordingly, herein we report the development of inflammation-specific AE and BER GRS and their associations with circulating hsCRP concentrations, individually and jointly with the DIS and LIS in two pooled cross-sectional studies.

## 2 Materials and Methods

### 2.1 Study Population

We pooled data from two, methodologically nearly identical, cross-sectional studies of patients going for outpatient, elective colonoscopies: Markers of Adenomatous Polyps (MAP) I, conducted 1994–1997 in Winston-Salem and Charlotte, North Carolina, and MAP II, conducted in Columbia, South Carolina in 2002. Both studies were conducted by the same principal investigator (RMB) using nearly identical protocols and questionnaires. Details of the MAP I and MAP II study protocols were previously published (Boyapati et al., 2004; Daniel et al., 2009). Briefly, participants were recruited from patients with no history of colorectal neoplasms who were scheduled for elective, outpatient colonoscopies at large gastroenterology clinics. Eligibility included English-speaking, 30–74 years of age, and capable of informed consent; exclusions included a history of colorectal adenomatous polyps, familial adenomatous polyposis, cancer other than non-melanoma skin cancer, known genetic syndromes associated with colonic neoplasia, and inflammatory bowel disease. The MAP I and MAP II consent rates were 67% and 76%, respectively, and the sample sizes were 526 and 267, respectively, yielding an initial pooled sample size of 793.

Each study was approved by the Institutional Review Board of the institution where it was conducted: Wake Forest University School of Medicine for MAP I and the University of South Carolina for MAP II. All participants provided informed consent, and the present data analyses were conducted using de-identified data. The MAP I and the MAP II studies hereinafter are referred to as the pooled MAP studies.

### 2.2 Data Collection and Laboratory Assays

Prior to colonoscopy visits, all study participants completed questionnaires on demographics, medical history, family history of colorectal cancer, lifestyle, self-reported anthropometrics (height, weight and waist and hip circumferences), diet [using a previously validated, semi-quantitative 153-item Willett food frequency questionnaire (FFQ) (Willett et al., 1985; MacIntosh et al., 1997)], and hormonal and reproductive history (in women). Physical activity was assessed using a modified Paffenbarger questionnaire (Paffenbarger et al., 1993), which queried usual lengths of times spent in specified moderate and vigorous activities on weekdays and weekends. The times spent in each activity category were summed, and the metabolic equivalents of task (METs) hours/week calculated; then the MET-hours/week from moderate and vigorous physical activities were summed. We calculated BMI as weight in kilograms divided by height in meters squared (kg/m^2^), and a waist:hip ratio, as waist circumference divided by hip circumference.

Participants completed their questionnaires at home and had fasting venous blood samples taken at their colonoscopy visit prior to the procedure. The blood samples were collected, handled, and stored in a manner allowing for genotyping and biomarker measurements. Blood was drawn into pre-chilled, red-coated Vacutainer tubes, which were immediately placed on ice and shielded from light, and taken to the laboratory. Tubes for serum and plasma were centrifuged under refrigeration, and aliquoted into amber-colored cryopreservation vials. The air in all aliquot vials was displaced with an inert gas (nitrogen in MAP I; argon in MAP II), and the vials were capped with O-ring screw caps. The aliquots were then immediately placed in −70°C freezers until analysis. hsCRP was measured *via* latex-enhanced immunonephelometry on a Behring nephelometer II (BN-II) analyzer (CV 4%; Behring Diagnostics). All hsCRP assays were performed at the Molecular Epidemiology and Biomarker Research Laboratory, University of Minnesota.

Tag single nucleotide polymorphisms (tagSNPs) that covered all SNPs with a minor allele frequency (MAF) >5% in two pathways—AE and DNA BER—were selected for genotyping and supplemented with candidate SNPs when available. For the AE genes, 6 SNPs were selected for *MnSOD*, 5 for *GTSP1*, and 11 for *CAT* (Supplementary Table S1). For the BER pathway genes, 11 SNPs were selected for *XRCC1*, 2 for *UNG*, 11 for *TDG*, 4 for *SMUG1*, 3 for *POLB*, 3 for *PNKP*, 6 for *OGG1*, 6 for *MUTYH*, 3 for *MPG*, 6 for *MBD4*, 15 for *LIG1*, 5 for *LIG3*, 1 for *FEN1*, and 3 for *APEX1* (Supplementary Table S2). Genotyping was conducted using the iPLEX Sequenom genotyping platform at the University of Minnesota Genomics Center, the core genotyping laboratory. The genotyping concordance for the selected SNPs in 64 pairs of blinded duplicate samples was ≥95% (Wang et al., 2017).

### 2.3 Data Analyses

#### 2.3.1 Exclusion Criteria

Prior to calculating the inflammation and genetic risk scores and conducting statistical analyses, we excluded from analysis participants missing or having extreme [>3 standard deviations (SD) of the study population mean] circulating hsCRP concentration values (*n* = 206), genotyping on >20% of the selected AE and BER genes (*n* = 184), and >15% of their food frequency questionnaire responses (*n* = 10); those who reported implausible total energy intakes (<600 or >6,000 kcal/day) (*n* = 10); those missing data on lifestyle factors (*n* = 12); non-White participants (*n* = 30) (because of population stratification and insufficient sample size for separate genetic analyses); and those with extreme (>10,000 IU/day) supplemental carotene intakes (*n* = 8), leaving a final sample size of 333 participants (MAP I = 187; MAP II = 146).

#### 2.3.2 Dietary and Lifestyle Inflammation Scores

Weighted DIS and LIS to represent the combined contributions of foods and beverages and lifestyle to systemic inflammation (Byrd et al., 2019) were originally developed in a heterogenous subset of the previously described Reasons for Geographic and Racial Differences in Stroke Study (REGARDS) cohort (Howard et al., 2005), and validated in three other study populations. Briefly, foods, beverages, and micronutrient supplements for the DIS were selected and grouped into 19 score components [18 whole foods and beverages plus a micronutrient supplement score (described below); Supplementary Table S3] *a priori* based on biological plausibility, prior literature, and applicability across different commonly used FFQs. The 18 whole foods and beverages included leafy greens and cruciferous vegetables, tomatoes, deep yellow or orange vegetables and fruits, legumes, refined grains and starchy vegetables, other vegetables, nuts, apples and berries, other fruits and real fruit juices, fish, poultry, red and organ meats, processed meats, high-fat dairy, low-fat dairy, other fat, added sugars, and coffee and tea. To account for supplemental micronutrients, a supplement score was calculated by ranking supplemental micronutrient intakes, based on the sex- and study-specific distribution, into tertiles. The tertiles were then assigned values of 0–2 and multiplied by +1 or −1 based on their literature-supported anti-inflammatory or pro-inflammatory contributions, respectively. The LIS comprised 4 components: physical activity, current smoking status, alcohol intake, and BMI (the detailed descriptions and rationales for inclusion are provided in Supplementary Table S3).

For the present study we calculated the DIS and LIS exactly as previously described (Byrd et al., 2019; Byrd et al., 2020). After calculating the components described above, we standardized each continuous food group, by sex, to a mean of 0 and SD of 1.0, and created dummy variables for the categorical variables in the LIS. Then, we multiplied the values by the previously reported weights (provided in Supplementary Table S3) and summed them to constitute the DIS and LIS. [Notes: a) The previously reported weights for the DIS and LIS components were based on the strengths of their multivariable-adjusted, individual associations with an inflammation biomarker score comprising four biomarkers of systemic inflammation in a diverse subset of the REGARDS cohort, and validated in three populations (Byrd et al., 2019). b) The biomarker score in REGARDS was calculated as the sum of normalized circulating concentrations of IL-6, IL-8, IL-10 (with a negative sign), and hsCRP]. A higher score represents a higher balance of pro-inflammatory to anti-inflammatory dietary or lifestyle exposures.

#### 2.3.3 Genetic Risk Scores

We developed AE and BER Genetic Risk Scores (GRS) as follows. First, we assessed all SNPs for Hardy-Weinberg equilibrium. We combined variant allele heterozygotes and homozygotes when there were ≤10 participants with either genotype. Then, for each genotype, we calculated and compared mean plasma hsCRP concentrations across genotypes using sex-and BMI-adjusted general linear models. For further perspective, from these results, we calculated proportional mean hsCRP concentration differences between the variant genotypes and the common homozygote as: (variant genotype-common homozygote/common homozygote x 100%) (Supplementary Tables S4, S5). We included SNPs in the AE GRS if the proportional mean differences in hsCRP concentrations were >5% with a *p*-value ≤0.05, or if the proportional mean differences in hsCRP concentrations were >10% with a *p*-value ≤0.15. Similarly, we included SNPs in the BER GRS if the proportional mean differences in hsCRP concentrations were >10% with a *p*-value ≤0.15. Based on these SNP selection criteria, for the AE GRS we included 1 SNP for *CAT* and 1 for *MnSoD* (Supplementary Table S6); and for the BER GRS we included 1 SNP for *MUTYH*, 1 for *SMUG1*, 3 for *TDG*, 1 for *UNG*, and 1 for *XRCC1* (Supplementary Table S7).

Next, for each SNP included in a GRS, we scored each variant allele 1 point and gave it a positive sign if the mean biomarker concentration was higher among those with the variant allele, and a negative sign if it was lower. Finally, we summed the values assigned to the genotypes to yield the respective GRS. A higher GRS indicates a higher balance of variant alleles directly associated with hsCRP relative to variant alleles inversely associated with hsCRP.

#### 2.3.4 Statistical Analyses

We summarized and compared the characteristics of the study participants across plasma hsCRP concentration tertiles using one-way ANOVA for continuous variables (transformed by the natural logarithm to meet normality assumptions when indicated) and chi-square tests for categorical variables.

We calculated and compared mean hsCRP concentrations across categories of exposure variables using multivariable general linear models, and because we transformed hsCRP concentrations by the natural logarithm, we report geometric means and their corresponding 95% confidence intervals (CI). We compared mean hsCRP concentrations across tertiles of the BER GRS, and dichotomized categories of the AE GRS (due to the small number of SNPs included). To assess potential interaction between the AE GRS and BER GRS in relation to mean hsCRP concentrations, we conducted a joint/combined (cross-classification) analysis in which participants in the joint lowest AE GRS category/BER GRS category were the reference group. Addition of covariates to the models for these main effects and interaction analyses negligibly affected the results, so we report only the crude results from these analyses. We also compared multivariable-adjusted mean hsCRP concentrations across DIS and LIS categories. For the DIS and LIS models, we selected covariates based on a combination of previous literature, biological plausibility, and whether inclusion/exclusion of potential covariates from the model affected the estimated proportional difference in the mean concentration of hsCRP between the highest and lowest score tertile by ≥10%. The covariates selected for the fully-adjusted models for both the DIS and LIS included sex, education (less than high school, high school degree, college graduate or higher), current hormone replacement therapy use (among women), and aspirin and/or other non-steroidal anti-inflammatory drug use (≥1/wk or <1/wk). The DIS models also included study, total energy intake, current smoking (yes/no), BMI (<25.0, 25.0–29.9, and ≥30.0 kg/m^2^), alcohol intake (none, moderate, heavy), and physical activity level (low, moderate, high); the LIS models also included the DIS (continuous). Finally, to assess potential modification of the inflammation scores-hsCRP associations by the two GRS, we further examined the adjusted associations of the inflammation scores with hsCRP concentrations stratified by dichotomized AE and BER GRS.

We conducted all statistical analyses using SAS version 9.4 software (SAS Institute, Inc. Cary, NC, United States), and considered a two-sided *p*-value ≤0.05 statistically significant.

## 3 Results

Selected characteristics of the participants according to hsCRP concentration tertiles are summarized in Table 1. Plasma hsCRP concentrations ranged from 0.14 to 32.67 μg/ml. Participants in the highest relative to the lowest hsCRP tertile were more likely to be female, a current smoker, and, if a woman, to take HRT; less likely to be formally educated and to have a heavy alcohol intake; and, on average, had a higher BMI, DIS, and LIS, and lower total vitamin C intake.

**TABLE 1:** Selected characteristics of participants (*n* = 333)^a^, by tertiles of plasma high sensitivity C-reactive protein (hsCRP), in the pooled MAP I and MAP II cross-sectional studies^b^.

Characteristics^a^	Plasma hsCRP tertiles			
	1	2	3	*p* ^c^
	0.14–1.63 μg/ml (*n* = 112)	1.64–4.40 μg/ml (*n* = 111)	4.41–32.67 μg/ml (*n* = 110)	
Demographics				
Age (years)	55.7 ± 8.5	57.2 ± 8.6	56.6 ± 8.4	0.46
Male (%)	58.9	48.7	35.5	0.002
College degree or higher (%)	45.7	27.7	14.8	< 0.001
Family history of CRC in a first degree relative (%)	19.6	33.3	33.0	0.06
Lifestyle				
Regular^d^ NSAID use (%)	32.1	32.4	33.0	0.99
Regular^d^ aspirin use (%)	42.7	36.0	30.0	0.14
HRT use in women (*n* = 174) (%)	39.1	63.2	62.0	0.02
Current smoker (%)	1.7	19.2	22.2	0.02
Body mass index (kg/m^2^)	26.2 ± 5.5	28.5 ± 6.1	30.5 ± 7.2	< 0.001
Waist:hip ratio	0.893 ± 0.102	0.922 ± 0.138	0.916 ± 0.094	0.13
Heavy alcohol intake^e^ (%)	10.7	9.0	5.5	0.01
Physical activity (METs/wk)^f^	293.5 ± 198.8	336.9 ± 217.3	321.0 ± 199.7	0.28
Circulating 25-OH-vitamin D_3_ (ng/ml)	28.2 ± 10.7	26.6 ± 12.2	24.9 ± 9.9	0.13
Dietary intakes				
Total energy (kcal/d)	1,794 ± 706	1,881 ± 678	1,883 ± 752	0.57
Total fat (% kcal/d)	31.7 ± 8.0	32.4 ± 7.0	33.1 ± 7.6	0.41
Total^g^ calcium (mg/1,000 kcal/d)	531 ± 345	497 ± 274	476 ± 320	0.42
Dietary fiber (g/1,000 kcal/d)	11.5 ± 4.7	11.1 ± 3.9	10.3 ± 3.3	0.08
Red & processed meats (servings/wk)	6.7 ± 5.2	7.4 ± 6.3	8.1 ± 5.6	0.18
Fruits & vegetables (servings/wk)	34.8 ± 20.9	35.8 ± 24.2	36.2 ± 25.2	0.90
Antioxidants:				
Total^g^ carotene (IU/1,000 kcal/d)	4,823 ± 3,392	4,403 ± 3,579	4,310 ± 3,413	0.50
Total^g^ lutein (mg/1,000 kcal/d)	1,761 ± 1,310	1,506 ± 1,060	1,629 ± 1,225	0.29
Total^g^ lycopene (mg/1,000 kcal/d)	2,830 ± 2,626	2,849 ± 2,221	2,712 ± 2,689	0.91
Total^g^ vitamin C (mg/1,000 kcal/d)	99 ± 218	71 ± 144	52 ± 140	0.005
Total^g^ vitamin E (mg/1,000 kcal/d)	38 ± 75	32 ± 98	33 ± 81	0.14
Total^g^ omega-3 fatty acid (g/1,000 kcal/d)	0.12 ± 0.10	0.11 ± 0.10	0.11 ± 0.12	0.78
Dietary flavonoids (mg/1,000 kcal/d)	264 ± 270	225 ± 219	222 ± 203	0.24
Dietary glucosinolates (mg/1,000 kcal/d)	9.6 ± 7.7	11.4 ± 11.5	11.6 ± 13.3	0.29
Supplemental selenium (mcg/1,000 kcal/d)	3.6 ± 10.3	5.3 ± 18.0	5.1 ± 20.6	0.43
Prooxidants:				
Total^g^ iron (mg/1,000 kcal/d)	13.2 ± 16.8	12.1 ± 11.3	11.5 ± 11.7	0.63
Dietary omega-6 fatty acids (g/1,000 kcal/d)	6.7 ± 2.8	6.5 ± 2.1	6.2 ± 2.2	0.27
Saturated fats (g/1,000 kcal/d)	11.3 ± 3.2	11.8 ± 2.9	12.5 ± 3.3	0.02
DIS^h^	0.6 ± 2.69	1.03 ± 2.88	1.63 ± 0.37	0.01
LIS^i^	0.14 ± 0.69	0.44 ± 0.69	0.72 ± 0.67	< 0.001

Abbreviations: CRC, colorectal cancer; DIS, dietary inflammation score; HRT, hormone replacement therapy; LIS, lifestyle inflammation score; MAP, Markers of Adenomatous Polyps; MET, metabolic equivalents of task; NSAID, non-steroidal anti-inflammatory drug.

a *n* = 331 for age, *n* = 332 for regular NSAID use, *n* = 246 for circulating 25-OH-vitamin D_3_, and *n* = 275 for dietary flavonoids and glucosinolates due to missing data.

b Data presented as mean (standard deviation) unless otherwise specified.

c *p*-values based on chi-square test for categorical variables and ANOVA for continuous variables (transformed by the natural logarithm to meet normality assumptions, when indicated).

d ≥ once per week.

e Heavy alcohol intake defined as >1 drink/day for women and >2 drinks/day for men.

f Moderate + vigorous physical activity.

g Total = dietary = supplemental.

h For construction of dietary inflammation score, see text; a higher score indicates a more proinflammatory diet.

i For construction of lifestyle inflammation score, see text; a higher score indicates a more proinflammatory lifestyle.

Mean plasma hsCRP concentrations according to AE and BER GRS categories are shown in Table 2. There was a dose-response pattern for increasing mean hsCRP concentrations across the BER GRS tertiles, and the mean hsCRP concentration among those in the highest relative to the lowest BER GRS tertile was, proportionately, statistically significantly 57.4% higher. Among those in the high relative to the low AE GRS category, the mean hsCRP concentration was estimated to be 13.9% higher, but the finding was not statistically significant. Adjustment for multiple covariates (e.g., sex, the DIS and the LIS) negligibly affected the estimates.

**TABLE 2:** Mean^a^ plasma high sensitivity C-reactive protein (hsCRP) concentrations according to categories of DNA base excision repair (BER) and antioxidant enzyme (AE) genetic risk scores (GRS), in the pooled MAP I and MAP II cross-sectional studies.

GRS, GRS categories	GRS category medians	Plasma hsCRP, µg/mL			
		*n*	Mean (95% CI)^a^	Prop. diff.^b^ (%)	*P* ^a^
BER GRS^c^					
Tertiles					
1	0	151	2.2 (1.9, 2.7)	Ref.	
2	1	113	3.0 (2.5, 3.7)	35.8	
3	2	69	3.5 (2.7, 4.6)	57.4	0.009
AE GRS^d^					
Low	−2	200	2.6 (2.2, 3.0)	Ref.	
High	3	133	2.9 (2.4, 3.6)	13.9	0.30

Abbreviations: AE, antioxidant enzyme; BER, base excision repair; CI, confidence interval; GRS, genetic risk score; hsCRP, high sensitivity C-reactive protein; MAP, Markers of Adenomatous Polyps; Prop. diff., proportional difference; Ref., reference.

a Geometric means, 95% confidence intervals, and *p*-values for mean differences from crude general linear models; unequal sample sizes in tertiles due to ranking ties; differences in the numbers of participants due to availability of blood samples for assays.

b Proportional difference calculated as (comparison group mean-reference group mean)/(reference group mean) x 100%.

c BER genetic risk score based on 7 SNPs in 5 BER genes; see complete list of genes and SNPs in the text and Supplementary Table S7; a higher GRS indicates a higher number of variant relative to common alleles.

d AE genetic risk score based on 2 SNPs in 2 AE genes; see complete list of genes and SNPs in the text and Supplementary Table S6; a higher GRS indicates a higher number of variant relative to common alleles.

The mean plasma hsCRP concentrations in the joint dichotomized AE/BER GRS categories are shown in Table 3. The lowest joint AE/BER GRS category was considered the reference group (the hypothesized lowest risk group). Although we found no statistically significant interaction between the AE and BER GRS, the mean hsCRP concentration was highest (62.7% higher) among those in the highest relative to the lowest joint AE/BER GRS category (*p* = 0.006). Adjustment for multiple covariates negligibly affected the estimates.

**TABLE 3:** Mean^a^ plasma high sensitivity C-reactive protein concentrations according to joint tertiles of antioxidant enzyme and DNA base excision repair genetic risk scores, in the pooled MAP I and MAP II cross-sectional studies (*n* = 333).

BER GRS categories^b^	AE GRS categories^c^									
	Low (< −1)					High (≥ −1)				
	n^d^	hsCRP means	(95% CI)	Prop. diff.^e^ (%)	*p*-values	n^d^	hsCRP means	(95% CI)	Prop. diff.^e^ (%)	*p*-values
Low (< 1)	90	2.0	(1.6, 2.6)	Ref.	—	61	2.6	(1.9, 3.4)	26.0	0.21
High (≥ 1)	110	3.1	(2.6, 3.9)	54.4	0.006	72	3.3	(2.5, 4.3)^f^	62.7	0.006

Abbreviations: AE, antioxidant enzyme; BER, base excision repair; CI, confidence interval; GRS, genetic risk score; hsCRP, high sensitivity C-reactive protein; MAP, Markers of Adenomatous Polyps; Prop. diff., proportional difference; Ref., reference.

a Geometric means, 95% confidence intervals, and *p*-values from crude general linear model.

b BER GRS based on 7 SNPs in 5 BER genes; see complete list of genes and SNPs in the text and Supplementary Table S5; categories based on median GRS.

c AE GRS based on 2 SNPs in 2 AE genes; see complete list of genes and SNPs in the text and Supplementary Table S4; categories based on median GRS.

d Unequal sample sizes in tertiles due to ranking ties; differences in the numbers of participants due to availability of blood samples for assays.

e Proportional difference calculated as (comparison group mean-reference group mean)/(reference group mean) x 100%.

f Continuous-by-continuous *P* for interaction = 0.65; categorical-by-categorical *P* for interaction = 0.47; categories based on median GRS.

We previously reported that in the larger (*n* = 423) pooled MAP studies population, which did not exclude participants missing genotyping data or who were not White, among those in the highest relative to the lowest DIS and LIS quartiles, multivariable-adjusted mean circulating hsCRP concentrations were 31.6% (*p* = 0.02) and 129% higher (*p* < 0.001), respectively (Byrd et al., 2020). In the present, more restricted pooled MAP studies population, among those in the highest relative to the lowest DIS and LIS tertiles, multivariable-adjusted mean circulating hsCRP concentrations were 18.5% (*p* = 0.66) and 120% higher (*p* < 0.001), respectively (Supplementary Table S8).

Multivariable-adjusted mean plasma hsCRP concentrations across DIS and LIS tertiles, stratified by dichotomized BER and AE GRS are shown in Table 4. We observed no strong or statistically significant evidence for effect modification of the DIS- and LIS-hsCRP associations by either GRS. However, there were some suggestions that the positive DIS-hsCRP association was slightly stronger among those with a high AE GRS, and that the positive LIS-hsCRP association was slightly stronger among those with a low AE GRS. Specifically, among those with a high AE GRS, the mean hsCRP concentration among those in the highest relative to the lowest DIS tertile was estimated to be 45.5% higher, whereas among those with a low AE GRS it was estimated to be 13.3% higher. Among those with a low AE GRS, the mean hsCRP concentration among those in the highest relative to the lowest LIS tertile was 194.3% higher, whereas among those with a high AE GRS it was estimated to be 68.6% higher.

**TABLE 4:** Multivariable-adjusted mean^a^ plasma high sensitivity C-reactive protein (hsCRP) concentrations (µg/ml) across tertiles of dietary (DIS) and lifestyle (LIS) inflammation scores, stratified by dichotomized DNA base excision repair (BER) and antioxidant enzymes (AE) genetic risk scores (GRS), in the pooled MAP I and MAP II cross-sectional studies.

GRS strata/tertiles of inflammation scores^b^	DIS					LIS				
	Tertile medians	n^c^	Mean^a^ (95% CI)	Prop. diff. (%)^d^	*P*	Tertile medians	n^c^	Mean^a^ (95% CI)	Prop. diff. (%)^d^	*P*
BER GRS^e^										
Low (< 1)										
1	−1.82	50	2.4 (1.5, 3.9)	Ref.		−0.41	50	1.8 (1.2, 2.6)	Ref.	
2	1.12	51	2.4 (1.6, 3.8)	1.2		0.50	52	2.5 (1.7, 3.6)	41.2	
3	3.29	50	3.2 (2.1, 5.1)	34.1	*0.36*	1.19	49	4.0 (2.7, 5.8)	123.7	0.001
High (≥ 1)										
1	−1.36	61	2.8 (1.9, 4.2)	Ref.		−0.41	59	2.1 (1.6, 2.9)	Ref.	
2	1.00	60	3.3 (2.2, 5.0)	17.3		0.50	61	3.1 (2.3, 4.1)	44.0	
3	4.06	61	3.3 (2.3, 4.9)	18.7	*0.65*	1.16	62	4.9 (3.6, 6.7)	130.9	< 0.001
*P*_*interaction*_ ^f^					*0.78*					0.80
AE GRS^g^										
Low (< −1)										
1	−1.48	66	2.7 (1.9, 3.9)	Ref.		−0.41	71	1.8 (1.4, 2.4)	Ref.	
2	1.07	67	3.3 (2.3, 4.7)	21.9		0.50	66	3.4 (2.5, 4.6)	85.7	
3	3.77	67	3.1 (2.2, 4.4)	13.3	*0.54*	1.16	63	5.4 (4.0, 7.3)	194.3	< 0.001
High (≥ −1)										
1	−1.72	44	2.7 (1.6, 4.4)	Ref.		−0.18	48	2.0 (1.4, 2.9)	Ref.	
2	1.04	45	2.0 (1.3, 3.2)	−23.1		0.71	43	2.3 (1.6, 3.4)	15.0	
3	3.55	44	3.9 (2.4, 6.3)	45.5	*0.06*	1.39	42	3.4 (2.3, 5.0)	68.6	0.08
*P*_*interaction*_ ^f^					*0.33*					0.56

Abbreviations: AE, antioxidant enzyme; BER, base excision repair; CI, confidence interval; DIS, dietary inflammation score; GRS, genetic risk score; hsCRP, high sensitivity C-reactive protein; LIS, lifestyle inflammation score; MAP, Markers of Adenomatous Polyps; Prop. diff., proportional difference; Ref., reference.

a Geometric means of plasma hsCRP (µg/ml), 95% confidence intervals, and *p*-values from general linear models; in the DIS models, adjusted for total energy intake, sex, education (less than high school, high school degree, college graduate or higher), current hormone replacement therapy use (among women), current smoking (yes/no), body mass index category, alcohol intake (non-, moderate-, heavy-drinker), physical activity level, study, and aspirin and/or other non-steroidal anti-inflammatory drug use (≥1/wk or <1/wk); in the LIS models, adjusted for sex, education (less than high school, high school degree, college graduate or higher), current hormone replacement therapy use (among women), aspirin and/or other non-steroidal anti-inflammatory drug use (≥1/wk or <1/wk), and DIS (continuous).

b For construction of scores, see text; higher scores indicate more proinflammatory diets or lifestyles.

c Unequal sample sizes in tertiles due to ranking ties; differences in the numbers of participants due to availability of blood samples for assays.

d Proportional difference calculated as (comparison group mean—reference group mean)/(reference group mean) x 100%.

e BER GRS based on 7 SNPs in 5 BER genes; see complete list of genes and SNPs in the text and Supplementary Table S7; categories based on GRS median.

g *P*_*interaction*_ from the interaction term for dichotomized GRS*inflammation score tertiles in general linear models.

f AE GRS based on 2 SNPs in 2 AE genes; see complete list of genes and SNPs in the text and Supplementary Table S6; categories based on GRS median.

## 4 Discussion

Our findings suggest that certain BER genotypes (*MUTYH*, *SMUG1*, *TDG*, *UNG*, and *XRCC1*) collectively may be associated with systemic inflammation, as reflected by circulating hsCRP concentrations. We found no definitive evidence that AE genotypes collectively are associated with systemic inflammation; however, plasma hsCRP concentrations were estimated to be modestly higher among individuals with more AE risk variants (i.e., had a higher AE GRS). We also found suggestions that the AE and BER GRS may i) modestly modify the associations of collective dietary and lifestyle exposures with systemic inflammation, and ii) interact with each other such that those with a higher relative to a lower joint BER/AE GRS may have modestly higher systemic inflammation. These findings from this preliminary investigation support further investigations in larger, general populations.

Previous basic science studies, reviewed extensively elsewhere (Sun, 1990; Seeberg et al., 1995; Mates, 2000; Di Virgilio, 2004; Kidane et al., 2014; Yang and Lee, 2015; Li and Chen, 2018), provide biological plausibility for investigating possible associations of AE and BER genotypes with inflammation. Briefly, higher levels of reactive oxygen and nitrogen species (RONS), from both exogenous and endogenous sources, damage cell structures and DNA, eliciting inflammatory responses (which in turn can increase RONS levels) and DNA BER, respectively. Antioxidant enzymes lower RONS levels, or neutralize their excessive cellular oxidation effects (Yang and Lee, 2015). As examples, of the genes included in our AE GRS, *CAT* defends against superoxide and hydrogen peroxide and constitutes a primary defense against oxidative stress (Röhrdanz and Kahl, 1998), and *MnSoD* prevents disruption of mitochondrial membrane potential and catalyzes dismutation of superoxide radicals (Röhrdanz and Kahl, 1998; Mäntymaa et al., 2000). As reviewed elsewhere (Kidane et al., 2014; Li and Chen, 2018), there is increasing evidence for interplay between DNA repair and inflammation. DNA damage is present during inflammation, accumulates during chronic inflammation, and DNA repair plays an important role in counteracting this damage (Kidane et al., 2014). RONS-induced DNA damage is generally repaired by the BER pathway (Wallace et al., 2012), and mutations in BER genes are associated with chronic inflammation. The DNA damage response (DDR) directly activates several transcription factors, such as NF-κB and interferon regulatory factors (IRFs) (McCool and Miyamoto, 2012). These transcription factors induce the expression of various immune genes, including those for inflammatory cytokines and chemokines. In addition, the DDR and oxidative stress induce the expression of several ligands for activating immune receptors. Although the exact mechanisms for these observations are not fully elucidated, possible mechanisms could include that accumulation of RONS-induced lesions (or associated mutations) in, for example, the mitochondrial genome, could lead to respiratory defects that increase intracellular ROS concentrations (Li and Chen, 2018); and oxidative DNA damage excision fragments could activate sensing programs such as cGAS/STING (Li and Chen, 2018), which may promote inflammatory outcomes. Evidence such as outlined above supports that DNA BER may play a role in controlling RONS levels, and that the efficiency of such control may affect RONS-induced inflammation. As examples of the polymorphic BER-related genes included in our BER GRS, *MUTYH* contributes to the repair of one of the most frequent and stable forms of oxidative damage by removing the mismatched 8-oxoG adenine (Sampson et al., 2005; Nielsen et al., 2011); *SMUG1* removes uracil from single- and double-stranded DNA in nuclear chromatin (Nilsen et al., 2001; Broderick et al., 2006); *TDG* has been implicated in DNA demethylation and could repair G/T and G/U mismatches *via* removing thymine and uracil moieties (He et al., 2011; Wu and Zhang, 2017); *UNG* repairs mutagenic G/U mismatches caused by deamination of cytosine (Krokan et al., 2001); and *XRCC1* plays a role in repairing single-stranded DNA breaks (Duell et al., 2000; Thompson and West, 2000). In summary, AE and BER genes reduce systemic inflammation *via* reducing RONS production, neutralizing oxidation, and repairing oxidative damage in humans.

Recently, GRS, also called polygenic risk scores (PRS), have been used as tools for investigating risk for various diseases (Wang et al., 2017; Chalmer et al., 2018; Korologou-Linden et al., 2019a; Korologou-Linden et al., 2019b; Leonenko et al., 2019; Mavaddat et al., 2019; Watt et al., 2019; Zheutlin et al., 2019; Li et al., 2020; Mosley et al., 2020; Sipeky et al., 2020), and support the concept of investigating associations of GRS with various outcomes. However, our study is the first to report associations of GRS with an inflammation biomarker. In epidemiologic studies, various GRS/PRS were reported to be associated with multiple diseases, including colorectal cancer (Wang et al., 2017), prostate cancer (Sipeky et al., 2020), breast cancer (Mavaddat et al., 2019; Watt et al., 2019), Alzheimer’s disease (Korologou-Linden et al., 2019a; Korologou-Linden et al., 2019b; Leonenko et al., 2019), and psychiatric diseases (Chalmer et al., 2018; Zheutlin et al., 2019). To our knowledge, there are no reported investigations of an AE GRS, and only one (Wang et al., 2017) of a BER GRS. That analysis used data pooled from three colonoscopy-based case-control studies (408 adenoma cases and 604 controls), and constructed a BER GRS based on 65 individual SNPs in 15 BER genes (Wang et al., 2017). That study’s findings suggested that colorectal adenoma risk among participants in the highest relative to the lowest tertile of that BER GRS was statistically significantly higher (odds ratio = 2.07, 95% CI = 1.26–3.40) (Wang et al., 2017). A cross-sectional study of hemodialysis patients (*n* = 167) investigated associations of BER genetic polymorphisms (3 SNPs; 2 for *MUTYH* and 1 for *OGG1*, but not the same as those in our study) with inflammation biomarkers (Cai et al., 2012). The findings suggested that two variant *MUTYH* genotypes, singly and in combination with an *OGG1* variant, were associated with higher IL-1β and IL-6 concentrations in that patient population (Cai et al., 2012). However, as noted, the authors investigated only three SNPs from BER genes and a GRS was not constructed (Cai et al., 2012).

Several studies investigated BER genetic variants as potential effect modifiers of associations of dietary and lifestyle exposures with colorectal adenoma risk (Corral et al., 2013; Wang et al., 2017), and support the concept of GRS as potential modifiers of associations of environmental exposures with various outcomes. However, our study is the first to report such potential effect modification in relation to an inflammation biomarker. Results from the three pooled case-control studies noted above suggested that the positive association of a higher balance of pro-over anti-oxidant dietary and lifestyle exposures with colorectal adenoma risk was stronger among those with more relative to fewer BER genetic variants (Wang et al., 2017). In a matched case-control study (677 adenoma cases and 691 controls), BER gene SNPs (although the SNPs investigated were not the same as those in our study) were estimated to be potential effect modifiers of the associations of colorectal adenoma with smoking (*MUTYH P*_*interaction*_ = 0.002, *OGG1 P*_*interaction*_ = 0.01, and *FEN1 P*_*interaction*_ = 0.01), alcohol (*LIG3 P*_*interaction*_ = 0.02), and dietary folate (*LIG3 P*_*interaction*_ = 0.02) (Corral et al., 2013).

Our study has several limitations. First, the GRS were calculated based on tagSNPs in only AE and BER genes in the present, relatively small study population, rather than *via* a genome-wide association study in a large population, and involved multiple comparisons. However, to our knowledge, our study is the first to report associations of GRS with an inflammation biomarker and provides support and preliminary data for future more definitive investigations. Second, our study population included only White participants who went for outpatient colonoscopy, which may limit the generalizability of our findings. Third, although our tagSNP approach comprehensively covered all common SNP variation in AE and BER genes, it was not possible to cover rare variation (MAF ≤5%); thus, potential influential SNPs may have been excluded. Fourth, we had measurements on only one biomarker of inflammation, hsCRP. A panel of biomarkers to represent multiple aspects of inflammation may more accurately represent systemic inflammation (Byrd et al., 2019), and as noted in Section 1, hsCRP has certain limitations. However, our study population was in general good health; we excluded those with inflammatory bowel disease, cancer, familial adenomatous polyposis, or extreme hsCRP values; assessed the use of NSAIDs as potential confounders and effect modifiers; and hsCRP measurements in such a population are highly correlated with those of other inflammation biomarkers (Byrd et al., 2019). Other limitations include the general limitations of using FFQs to assess diet, such as recall error, limited food choices, and issues regarding capturing seasonal intake patterns. However, we used a previously validated FFQ (Willett et al., 1985), and in our study, recall error would be expected to be non-differential (participants reported dietary and lifestyle exposures before hsCRP measurements), which would be expected to attenuate the DIS and LIS results.

Strengths of our study include the collection and assessment of extensive dietary, lifestyle, and medical data as potential confounding factors, the high quality of the laboratory measurements, and the inclusion of both men and women. Also, to our knowledge, our study is the first to report i) associations of AE and BER GRS with a biomarker of inflammation in humans, and ii) AE and BER GRS as potential effect modifiers of the associations of collective dietary and lifestyle exposures with a biomarker of inflammation.

In conclusion, our findings, taken together with previous literature, suggest that, collectively, genotypes of DNA BER genes may be associated with systemic inflammation in humans. Our study serves as a pilot study that supports further investigations of inflammation-specific BER and AE GRS, individually and in interaction with each other and dietary and lifestyle inflammation scores, in larger, general populations.

## Data Availability

The data analyzed in this study is subject to the following licenses/restrictions: Data from this study are available upon application to the corresponding author. Requests to access these datasets should be directed to Roberd M. Bostick, rmbosti@emory.edu.
