# Supplementary material for: Associations of DNA Base Excision Repair and Antioxidant Enzyme Genetic Risk Scores with Biomarker of Systemic Inflammation
Source: Front Aging. 2022 May 4;3:897907. doi: 10.3389/fragi.2022.897907 (PMC9632613; doi:10.3389/fragi.2022.897907)
Supplement: Supplementary file 1 [file DataSheet1.docx]

***Supplemental Materials***

**Supplemental Table 1.** Antioxidant enzyme genes investigated

| **Gene** | **SNP rs ID** | **RefSNP alleles (strand direction)** | **Minor allele**^a^ |
| --- | --- | --- | --- |
| *MnSOD* | rs4880 | C/T (REV) | G |
| *MnSOD* | rs5746151 | A/G (REV) | T |
| *MnSOD* | rs5746136 | A/G (REV) | T |
| *MnSOD* | rs2842980 | A/T (REV) | A |
| *MnSOD* | rs6917589 | C/T (FWD) | C |
| *MnSOD* | rs8031 | A/T (REV) | T |
|  |  |  |  |
| *GSTP1* | rs1695 | A/G (FWD) | G |
| *GSTP1* | rs1138272 | C/T (FWD) | T |
| *GSTP1* | rs4147581 | C/G (FWD) | G |
| *GSTP1* | rs749174 | C/T (REV) | A |
| *GSTP1* | rs762803 | A/C (FWD) | A |
|  |  |  |  |
| *CAT* | rs1001179 | A/G (REV) | T |
| *CAT* | rs7947841 | A/G (FWD) | A |
| *CAT* | rs499406 | A/C/G (REV) | T |
| *CAT* | rs566979 | A/G/T (REV) | C |
| *CAT* | rs16925614 | C/T (FWD) | T |
| *CAT* | rs11032703 | C/T (FWD) | T |
| *CAT* | rs11604331 | A/G (FWD) | G |
| *CAT* | rs525938 | A/G (REV) | C |
| *CAT* | rs7104301 | A/G (FWD) | G |
| *CAT* | rs12272630 | C/G (FWD) | C |
| *CAT* | rs7943316 | A/T (FWD) | T |

Abbreviations: FWD, forward; ID, identifier; REV, reverse; SNP, single nucleotide polymorphism

^a^ Data from 1000 Genomes Project

**Supplemental Table 2.** DNA base excision repair pathway genes investigated

| **Gene** | **SNP rs ID** | **RefSNP alleles (strand direction)** | **Minor allele**^a^ |
| --- | --- | --- | --- |
| *XRCC1* | rs939461 | A/C (FWD) | C |
| *XRCC1* | rs3213247 | G/T (REV) | A |
| *XRCC1* | rs939460 | A/G (FWD) | A |
| *XRCC1* | rs25487 | A/G (REV) | T |
| *XRCC1* | rs25489 | A/C/G (REV) | T |
| *XRCC1* | rs1001581 | C/T (FWD) | T |
| *XRCC1* | rs2307191 | C/T (REV) | A |
| *XRCC1* | rs3213403 | A/G (REV) | C |
| *XRCC1* | rs915927 | A/C/G/T (REV) | C |
| *XRCC1* | rs3213255 | C/T (REV) | G |
| *XRCC1* | rs25496 | C/T (REV) | G |
|  |  |  |  |
| *UNG* | rs3219245 | G/T (FWD) | T |
| *UNG* | rs246079 | A/G (FWD) | A |
|  |  |  |  |
| *TDG* | rs3829301 | A/C (FWD) | C |
| *TDG* | rs4135113 | A/G/T (FWD) | A |
| *TDG* | rs2629768 | A/G (REV) | T |
| *TDG* | rs4135064 | C/T (FWD) | T |
| *TDG* | rs322107 | C/T (REV) | A |
| *TDG* | rs4135061 | A/G (FWD) | G |
| *TDG* | rs4135081 | A/G (FWD) | G |
| *TDG* | rs322109 | A/C/G (REV) | C |
| *TDG* | rs4135093 | C/T (FWD) | C |
| *TDG* | rs4135094 | C/T (FWD) | C |
| *TDG* | rs167715 | C/T (REV) | G |
|  |  |  |  |
| *SMUG1* | rs2233920 | G/T (REV) | A |
| *SMUG1* | rs3136386 | C/G (REV) | C |
| *SMUG1* | rs971 | C/T (FWD) | T |
| *SMUG1* | rs2279402 | C/T (REV) | G |
|  |  |  |  |
| *POLB* | rs2979896 | G/T (REV) | C |
| *POLB* | rs3136811 | C/G (FWD) | G |
| *POLB* | rs3136797 | C/G (FWD) | G |
|  |  |  |  |
| *PNKP* | rs3739206 | G/T (REV) | C |
| *PNKP* | rs2257103 | C/T (FWD) | T |
| *PNKP* | rs3739186 | A/T (REV) | T |
|  |  |  |  |
| *OGG1* | rs125701 | A/G (FWD) | A |
| *OGG1* | rs1805373 | A/C/G (FWD) | A |
| *OGG1* | rs2072668 | C/G (FWD) | G |
| *OGG1* | rs3219008 | A/G (FWD) | G |
| *OGG1* | rs159153 | C/T (FWD) | C |
| *OGG1* | rs293795 | C/T (REV) | G |
|  |  |  |  |
| *MUTYH* | rs3219476 | G/T (REV) | A |
| *MUTYH* | rs3219484 | A/G (REV) | T |
| *MUTYH* | rs3219494 | A/G (REV) | T |
| *MUTYH* | rs3219463 | A/G (REV) | T |
| *MUTYH* | rs3219489 | C/G (REV) | T |
| *MUTYH* | rs3219493 | C/G (REV) | G |
|  |  |  |  |
| *MPG* | rs3176415 | A/G (FWD) | G |
| *MPG* | rs2541622 | C/G/T (REV) | A |
| *MPG* | rs3176424 | A/G (FWD) | G |
|  |  |  |  |
| *MBD4* | rs3138360 | A/G (REV) | T |
| *MBD4* | rs10342 | A/G/T (REV) | T |
| *MBD4* | rs2005618 | C/T (REV) | G |
| *MBD4* | rs2311394 | C/T (REV) | G |
| *MBD4* | rs2307293 | C/G (REV) | G |
| *MBD4* | rs3138326 | A/T (REV) | A |
|  |  |  |  |
| *LIG1* | rs419664 | A/G/T (REV) | A |
| *LIG1* | rs156641 | A/G (REV) | T |
| *LIG1* | rs2288881 | A/G (REV) | T |
| *LIG1* | rs3730947 | A/G (REV) | T |
| *LIG1* | rs3731037 | C/T (REV) | A |
| *LIG1* | rs411073 | C/T (REV) | A |
| *LIG1* | rs3730908 | C/T (REV) | A |
| *LIG1* | rs20579 | C/G/T (REV) | A |
| *LIG1* | rs3730881 | C/T (REV) | A |
| *LIG1* | rs3730914 | C/T (REV) | A |
| *LIG1* | rs3731003 | C/T (REV) | A |
| *LIG1* | rs3730837 | A/G (REV) | C |
| *LIG1* | rs274862 | C/T (FWD) | C |
| *LIG1* | rs3730912 | A/C (REV) | T |
| *LIG1* | rs20580 | A/C (REV) | G |
|  |  |  |  |
| *LIG3* | rs3135974 | A/G (FWD) | A |
| *LIG3* | rs3135998 | A/G (FWD) | A |
| *LIG3* | rs3135989 | G/T (FWD) | G |
| *LIG3* | rs3135967 | A/G (FWD) | G |
| *LIG3* | rs2074516 | C/G (REV) | G |
|  |  |  |  |
| *FEN1* | rs412334 | A/G (REV) | T |
|  |  |  |  |
| *APEX1* | rs3136814 | A/C (FWD) | C |
| *APEX1* | rs1130409 | A/G/T (FWD) | G |
| *APEX1* | rs1760944 | A/C (REV) | T |

Abbreviations: FWD, forward; ID, identifier; REV, reverse; SNP, single nucleotide polymorphism

^a^ Data from 1000 Genomes Project

**Supplemental Table 3.** Components of the DIS and LIS, their general descriptions, rationale for inclusion, and assigned weights^a^ in the pooled MAP I and MAP II studies

| **Components** | **Descriptions** | **Rationale** | **Weights**^a^ |
| --- | --- | --- | --- |
| **LIS components**^b^ | |  |  |
| Heavily physically active | Individuals in the study population highest vs. lowest tertile of MET-hours per week | Physical activity improves systemic plasma antioxidant capacity (increases adaptive responses to oxidative stress), increases concentrations of anti-inflammatory cytokines, and lowers vascular wall inflammation (Gomez-Cabrera et al., 2008; Calder et al., 2011) | -0.41 |
| Moderately physically active | Individuals in the study population middle vs. lowest tertile of MET-hours per week | Mechanisms similar to those described above | -0.18 |
| Heavy drinker | Heavy (defined as > 7 drinks/week for women, > 14 drinks/week drinks for men) vs. non-drinker | Heavy alcohol intake results in oxidative stress via oxidation of ethanol to acetaldehyde (Wu et al., 2006; Das and Vasudevan, 2007) | 0.30 |
| Moderate drinker | Heavy (defined as 1 – 7 drinks/week for women, 1 – 14 drinks/week drinks for men) vs. non-drinker | A metabolite of ethanol is acetate, which can acutely lower proinflammatory free fatty acid concentrations; moderate alcohol intake increases serum adiponectin concentrations (an anti-inflammatory inflammation biomarker) (Mathews et al., 2015) and inhibits IL-6 production and activity (McCarty, 1999) | -0.66 |
| Current smoker | currently smokes tobacco vs. does not currently smoke tobacco | Toxins injure tissues, upregulating cytokines and acute-phase reactants (van der Vaart et al., 2004) | 0.50 |
| Obese BMI | Obese (defined as BMI ≥ 30 kg/m^2^) vs. normal BMI | Adipose tissue synthesizes and releases proinflammatory adipokines, such as PAI-1 and TNF-α (Furukawa et al., 2004; Calder et al., 2011) | 1.57 |
| Overweight BMI | Overweight (defined as BMI 25 – 29.99 kg/m^2^) vs. normal BMI | Mechanisms similar to those described above | 0.89 |

| **DIS components**^c^ |  |  |  |
| --- | --- | --- | --- |
| Leafy greens & cruciferous vegetables | Kale, spinach, broccoli, Brussels sprout, cabbage or coleslaw, cauliflower, and iceberg, head lettuce, romaine, or leaf lettuce | Contain variety of potent antioxidants (e.g., β-carotene, folacin, magnesium, calcium, glucosinolates, isothiocyanates, lutein, and indoles); contain flavonoids and polyphenols, which activate the transcription factor, nuclear factor-erythroid 2 (NF-E2)-related factor 2 (Nrf2), which plays a key role in cellular protection against oxidative stress and inflammation (Sommerburg et al., 1998; Brown and Hu, 2001; Guardia et al., 2001; Obeid et al., 2011; Wang et al., 2013; Johnson, 2014; Du et al., 2015; Nidhi et al., 2015; Kelly et al., 2016; Hussain et al., 2017; Johnson et al., 2018) | -0.14 |
| Tomatoes | Tomatoes, tomato juice, tomato sauce, salsa, and ketchup | Contain β-carotene, vitamin C, and lycopene, the latter of which is a potent singlet oxygen quencher and one of the most powerful antioxidants among the natural carotenoids (Rao, 2002; Jacob et al., 2008; Markovits et al., 2009; Burton-Freeman and Sesso, 2014) | -0.78 |
| Deep yellow or orange vegetables & fruit | Cantaloupe, peaches, and carrots | Contain pro-vitamin A carotenoids (e.g., β-carotene and α-carotene), which have a conjugated double-bond structure making them strong antioxidants (Calder, 2010) | -0.57 |
| Legumes | String beans, peas, lima beans, lentils, and other beans | Contain folacin, iron, isoflavones, protein, vitamin B6, and have a high antioxidant capacity; rich in fiber, which is associated with beneficial alterations to the gut microbiota, reducing immune response in the gut (Brown and Hu, 2001; Hartman et al., 2010; Zitvogel et al., 2017) | -0.04 |
| Refined grains & starchy vegetables | Cold or cooked breakfast cereal, white or dark bread, bagels, English muffins, rolls, cornbread, white rice, pasta, pancakes or waffles, sweet potatoes or yams, potato chips, crackers, tortillas, popcorn, pretzels, cookies, brownies, doughnuts, cake, pie, sweet rolls or coffee cakes, and French fried, scalloped, baked, boiled, or mashed potatoes | Sparse in nutrients; some processed grains contain emulsifiers, which potentially break down mucin in the gut leading to inflammation (Chassaing et al., 2015); and induce hyperglycemia (mechanisms described similar to those described above in ‘Added Sugars’) | 0.72 |
| Other vegetables | Beets, celery, eggplant, garlic, green peppers, mushrooms, and onions | Contain antioxidants and polyphenols with similar mechanisms to those described above | -0.16 |
| Nuts | Peanuts, peanut butter, and other nuts | Contain Ω -3 fatty acids (Simopoulos, 2002; Giugliano et al., 2006; Casas-Agustench et al., 2010; Wall et al., 2010) (mechanisms similar to those described above in ‘Fish’) and contain *l*-arginine (Brown and Hu, 2001), which improves endothelium-dependent dilation (precursor of the endogenous vasodilator nitric oxide) and decreases platelet aggregation and monocyte adhesion (Brown and Hu, 2001) | -0.44 |
| Apples & berries | Fresh apples or pears, applesauce, apple juice or cider, strawberries, and blueberries | Contain flavonoids (e.g., anthocyanins, quercetin, and phenolic acids) that suppress pro-inflammatory cytokine production and are powerful antioxidants; potentially increase postprandial plasma antioxidant capacity (Prior et al., 2007; Codoñer-Franch et al., 2013; Espley et al., 2014) | -0.65 |
| Other fruits & real fruit juices | Pineapples, honeydew, watermelon, grapes, prunes, oranges, orange juice, grapefruit, grapefruit juice, and other real fruit juices | Contain antioxidants (e.g., flavonoids, such as hesperidin, naringenin, neohesperidin, limonene, vitamin C, β-cryptoxanthin, plant sterols, salicylates, naringin, nobelitin, and narirutin) with similar mechanisms to those described above (Guardia et al., 2001; Knekt et al., 2002; Böhm et al., 2007; Ghanim et al., 2007; Hale et al., 2010; Fortis-Barrera et al., 2013; Alam et al., 2014; Jia et al., 2015; Sharma et al., 2015) | -0.16 |
| Fish | Canned tuna fish or salmon, dark meat fish, other fish, and breaded fish cakes or fish sticks | Contain Ω-3 fatty acids, which compete with pro-inflammatory Ω-6 fatty acids by synthesizing eicosanoids and suppress the capacity of monocytes to synthesize IL-1β and TNF- α (Simopoulos, 2002; Giugliano et al., 2006; Calder, 2010) | -0.08 |
| Poultry | Chicken and turkey with and without skin | Inversely associated with inflammation markers (van Woudenbergh et al., 2012), contain low amounts of saturated fat (van Bussel et al., 2015), and contain *l*-arginine (see mechanisms in ‘Nuts’) | -0.45 |
| Red & organ meats | Beef, pork, lamb, liver, and other organ meats | Contain heme iron (see above); contain Ω-6 fatty acids and saturated fat (see mechanisms in ‘Fats’ above) | 0.02 |
| Processed meats | Bacon, salami, bologna, other processed meats, and beef, pork, chicken, or turkey hot dogs | Contain heme iron, which increases the bioavailability of iron, which in turn increases oxidative stress; contain higher saturated fat contents, Ω-6 fatty acids (see ‘Fats’), and additives, such as nitrites, with suspected pro-inflammatory properties (van Woudenbergh et al., 2012; White and Collinson, 2013) | 0.68 |
| Added sugars | Soda, punch, lemonade, fruit drinks, chocolate candy bars, other mixed candy bars, candy without chocolate, jams, jellies, preserves, and syrup or honey | Sparse in nutrients; induce postprandial hyperglycemia, which act as stressful stimuli through subsequent repeated mild postprandial hypoglycemia (Kallio et al., 2007) and reduce nitric oxide availability (plays role in regulation of inflammatory response (Giugliano et al., 2006); elevate pro-inflammatory free fatty acid levels (Guzik et al., 2003); produce oxidative stress through oxidation of membrane lipids, proteins, lipoproteins, and DNA (Ludwig, 2002) | 0.56 |
| Coffee & tea | Coffee (decaf and regular) and tea (herbal and non-herbal) | Tea contains flavonoids and antioxidants (e.g., epicatechin and quercetin) (Dower et al., 2015); coffee contains phytochemicals and antioxidants, such as javamide; ﻿both coffee and tea contain varying amounts of caffeine which inhibit secretion of IL-1β induced by adenine and N4-acetylcytidine (Zitvogel et al., 2017; Park, 2018) | -0.25 |
| High-fat dairy | Whole milk, ice cream, cream cheese, full-fat cheeses, and sour cream | Contains calcium, which binds bile acids and free fatty acids, decreasing oxidative damage in the gut; dairy fat contains fatty acids with potential inflammation-reducing properties, such as CLA, *cis-* and *trans-*palmitoleic acid, butyric acid, phytanic acid, and alpha-linolenic acid (Govers et al., 1996; Ludwig, 2002; Dash et al., 2013) | -0.14 |
| Low-fat dairy | Low-fat yogurt, low-fat cottage or ricotta cheese, other low-fat cheeses, and skim, 1%, 2%, or low-fat milk | Similar mechanisms to high-fat dairy (see mechanisms above), with lower fat content | -0.12 |
| Other fats | Mayonnaise, margarine, and butter | Contain Ω-6 fatty acids and saturated fats (see ‘red and organ meats’ above) | 0.31 |
| Supplement score^d^ | Ranked score of supplements, including: vitamins A, B1, B12, B6, C, D, and E; and β-carotene, folate, niacin, riboflavin, calcium, iron, magnesium, selenium, and zinc | Comprises micro-nutrients, minerals, and vitamins solely from supplement intakes, some with similar mechanisms to those described above (e.g., iron as pro-oxidant, vitamins A, C, and E as antioxidants) | -0.80 |

Abbreviations: CLA, conjugated linoleic acid; BMI, body mass index; DIS, dietary inflammation score; hsCRP, high-sensitivity C-reactive protein; LIS, lifestyle inflammation score; MET, metabolic equivalent of task; Nrf2, nuclear factor-erythroid 2 (NF-E2)-related factor 2; NSAID, nonsteroidal anti-inflammatory drug; PAI-1, plasminogen activator inhibitor-1; REGARDS, Reasons for Geographic and Racial Differences in Stroke Study

^a^ Weights are β-coefficients from multivariable linear regression models conducted in the REGARDS case-cohort sample (n = 639), representing the average change in a summary inflammation biomarker score [sum of z scores for hsCRP, IL-6, IL-8, IL-10 (the latter with a negative sign)] per 1 SD increase in a dietary component or the presence of lifestyle component. A negative weight indicates that a component has an anti-inflammatory effect, a positive weight indicates that a component has a proinflammatory effect. Covariates in the final model included: age, sex, race (black or white), education (high school graduate or less vs. some college or more), region (stroke belt, stroke buckle, or other region in the United States), a comorbidity score (comprises a history of cancer, heart disease, diabetes mellitus, or chronic kidney disease), regular use of aspirin, other NSAIDs, or lipid-lowering medications (≥ twice/wk), hormone replacement therapy (among women), total energy intake (kcal/d), season of baseline interview (spring, summer, fall, or winter); and all the dietary/lifestyle components in the DIS and LIS.

^b^ All lifestyle components were dummy variables, coded as “1” for the nonreferent category and “0” for the referent category.

^c^ Dietary components were standardized to the study population sample, by sex, to a mean of zero and SD of 1.

^d^ All vitamin and mineral supplement intakes measured (from multivitamin/mineral and individual supplements) were ranked into tertiles of intake and assigned a value of 0 (low or no intake), 1, or 2 (highest intake) for hypothesized anti-inflammatory supplements (i.e., all but iron), and 0 (low or no intake), -1, or -2 (highest intake) for hypothesized pro-oxidant supplements (i.e., iron), and then the values were summed.

**Supplemental Table 4.** Distributions of antioxidant enzyme genotypes, and mean plasma high sensitivity C-reactive protein concentrations according to the genotypes, in the pooled MAP I and MAP II cross-sectional studies (n = 333)^a^

| **Gene** | **SNP** | **Genotype** | **Weight** | **n** | **HWE** | | |  | **Geometric means** | | | **Prop. diff.**^c^ **(%)** | ***P*-value** |
| --- | --- | --- | --- | --- | --- | --- | --- | --- | --- | --- | --- | --- | --- |
|  |  |  |  |  | **Expected n**^b^ | **χ^2^** | ***P-value*** |  | **Mean** | **95% CL** | |  |  |
|  |  |  |  |  |  |  |  |  |  | **LL** | **UL** |  |  |
| ***CAT*** | **rs1001179** |  |  |  |  |  |  |  |  |  |  |  |  |
|  |  | **Missing** | 0 | 6 |  |  |  |  |  |  |  |  |  |
|  |  | **GG** | 0 | 190 | 193.4 |  |  |  | 2.6 | 2.3 | 3.1 |  |  |
|  |  | **GA** | 1 | 123 | 116.1 |  |  |  | 2.8 | 2.4 | 3.4 |  |  |
|  |  | **AA** | 2 | 14 | 17.4 | 1.14 | *0.29* |  | 2.2 | 1.3 | 3.8 | -16.4 | *0.62* |
|  |  |  |  |  |  |  |  |  |  |  |  |  |  |
| ***CAT*** | **rs7947841**^d^ |  |  |  |  |  |  |  |  |  |  |  |  |
|  |  | **Missing** | 0 | 6 |  |  |  |  |  |  |  |  |  |
|  |  | **GG** | 0 | 290 | 289.2 |  |  |  | 2.6 | 2.3 | 3.0 |  |  |
|  |  | **GA** | 1 | 35 | 36.7 |  |  |  | 3.3 | 2.3 | 4.6 |  |  |
|  |  | **AA** | 2 | 2 | 1.2 | 0.68 | *0.41* |  | 1.5 | 0.4 | 6.5 | -42.1 | *0.69* |
|  |  | **GA + AA** |  | 37 |  |  |  |  | 3.1 | 2.2 | 4.4 | 18.6 | *0.35* |
|  |  |  |  |  |  |  |  |  |  |  |  |  |  |
| ***CAT*** | **rs499406** |  |  |  |  |  |  |  |  |  |  |  |  |
|  |  | **Missing** | 1 | 6 |  |  |  |  |  |  |  |  |  |
|  |  | **GG** | 0 | 119 | 111.0 |  |  |  | 2.5 | 2.1 | 3.0 |  |  |
|  |  | **GA** | 1 | 143 | 159.0 |  |  |  | 2.9 | 2.5 | 3.5 |  |  |
|  |  | **AA** | 2 | 65 | 57.0 | 3.33 | *0.07* |  | 2.6 | 2.0 | 3.4 | 4.9 | *0.46* |
|  |  |  |  |  |  |  |  |  |  |  |  |  |  |
| ***CAT*** | **rs566979** |  |  |  |  |  |  |  |  |  |  |  |  |
|  |  | **Missing** | 1 | 6 |  |  |  |  |  |  |  |  |  |
|  |  | **TT** | 0 | 130 | 131.0 |  |  |  | 2.7 | 2.3 | 3.3 |  |  |
|  |  | **TG** | 1 | 154 | 151.9 |  |  |  | 2.6 | 2.2 | 3.0 |  |  |
|  |  | **GG** | 2 | 43 | 44.0 | 0.06 | *0.81* |  | 2.9 | 2.2 | 4.0 | 8.2 | *0.74* |
|  |  |  |  |  |  |  |  |  |  |  |  |  |  |
| ***CAT*** | **rs16925614**^d^ |  |  |  |  |  |  |  |  |  |  |  |  |
|  |  | **Missing** | 0 | 7 |  |  |  |  |  |  |  |  |  |
|  |  | **CC** | 0 | 231 | 232.8 |  |  |  | 2.8 | 2.5 | 3.2 |  |  |
|  |  | **CT** | 1 | 89 | 85.4 |  |  |  | 2.3 | 1.9 | 2.9 |  |  |
|  |  | **TT** | 2 | 6 | 7.8 | 0.59 | *0.44* |  | 2.1 | 0.9 | 4.8 | -25.4 | *0.25* |
|  |  | **CT + TT** |  | 91 |  |  |  |  | 2.3 | 1.9 | 2.8 | -18.9 | *0.10* |
|  |  |  |  |  |  |  |  |  |  |  |  |  |  |
| ***CAT*** | **rs11032703**^d^ |  |  |  |  |  |  |  |  |  |  |  |  |
|  |  | **Missing** | 0 | 7 |  |  |  |  |  |  |  |  |  |
|  |  | **CC** | 0 | 256 | 257.1 |  |  |  | 2.6 | 2.3 | 3.0 |  |  |
|  |  | **CT** | 1 | 67 | 64.8 |  |  |  | 3.0 | 2.4 | 3.9 |  |  |
|  |  | **TT** | 2 | 3 | 4.1 | 0.37 | *0.54* |  | 4.3 | 1.3 | 14.1 | 65.8 | *0.40* |
|  |  | **CT + TT** |  | 70 |  |  |  |  | 3.1 | 2.4 | 3.9 | 18.7 | *0.22* |
|  |  |  |  |  |  |  |  |  |  |  |  |  |  |
| ***CAT*** | **rs11604331** |  |  |  |  |  |  |  |  |  |  |  |  |
|  |  | **Missing** | 1 | 6 |  |  |  |  |  |  |  |  |  |
|  |  | **AA** | 0 | 140 | 136.1 |  |  |  | 2.5 | 2.1 | 2.9 |  |  |
|  |  | **AG** | 1 | 142 | 149.7 |  |  |  | 2.9 | 2.4 | 3.4 |  |  |
|  |  | **GG** | 2 | 45 | 41.1 | 0.87 | *0.35* |  | 2.8 | 2.0 | 3.8 | 11.7 | *0.47* |
|  |  |  |  |  |  |  |  |  |  |  |  |  |  |
| ***CAT*** | **rs525938** |  |  |  |  |  |  |  |  |  |  |  |  |
|  |  | **Missing** | 0 | 6 |  |  |  |  |  |  |  |  |  |
|  |  | **AA** | 0 | 164 | 166.0 |  |  |  | 2.7 | 2.3 | 3.1 |  |  |
|  |  | **AG** | 1 | 138 | 134.0 |  |  |  | 2.8 | 2.3 | 3.3 |  |  |
|  |  | **GG** | 2 | 25 | 27.0 | 0.30 | *0.59* |  | 2.4 | 1.6 | 3.6 | -9.3 | *0.81* |
|  |  |  |  |  |  |  |  |  |  |  |  |  |  |
| ***CAT*** | **rs7104301** |  |  |  |  |  |  |  |  |  |  |  |  |
|  |  | **Missing** | 0 | 7 |  |  |  |  |  |  |  |  |  |
|  |  | **AA** | 0 | 170 | 173.8 |  |  |  | 2.8 | 2.4 | 3.3 |  |  |
|  |  | **AG** | 1 | 136 | 128.5 |  |  |  | 2.5 | 2.1 | 2.9 |  |  |
|  |  | **GG** | 2 | 20 | 23.8 | 1.11 | *0.29* |  | 2.8 | 1.8 | 4.4 | -0.9 | *0.48* |
|  |  |  |  |  |  |  |  |  |  |  |  |  |  |
| ***CAT*** | **rs12272630**^d^ |  |  |  |  |  |  |  |  |  |  |  |  |
|  |  | **Missing** | 0 | 6 |  |  |  |  |  |  |  |  |  |
|  |  | **GG** | 0 | 312 | 310.2 |  |  |  | 2.7 | 2.4 | 3.0 |  |  |
|  |  | **GC** | 1 | 13 | 16.6 |  |  |  | 3.1 | 1.8 | 5.5 |  |  |
|  |  | **CC** | 2 | 2 | 0.2 | 15.10 | *< 0.01* |  | 1.3 | 0.3 | 5.5 | -52.9 | *0.52* |
|  |  | **GC + CC** |  | 15 |  |  |  |  | 2.8 | 1.6 | 4.7 | 3.2 | *0.91* |
|  |  |  |  |  |  |  |  |  |  |  |  |  |  |
| ***CAT*** | **rs7943316** |  |  |  |  |  |  |  |  |  |  |  |  |
|  |  | **Missing** | 1 | 6 |  |  |  |  |  |  |  |  |  |
|  |  | **TT** | 0 | 139 | 143.3 |  |  |  | 2.7 | 2.3 | 3.2 |  |  |
|  |  | **TA** | 1 | 155 | 146.3 |  |  |  | 2.8 | 2.4 | 3.3 |  |  |
|  |  | **AA** | 2 | 33 | 37.3 | 1.15 | *0.28* |  | 2.2 | 1.5 | 3.1 | -19.2 | *0.42* |
|  |  |  |  |  |  |  |  |  |  |  |  |  |  |
| ***GSTP1*** | **rs4147581** |  |  |  |  |  |  |  |  |  |  |  |  |
|  |  | **Missing** | 1 | 15 |  |  |  |  |  |  |  |  |  |
|  |  | **CC** | 0 | 93 | 90.9 |  |  |  | 2.6 | 2.1 | 3.2 |  |  |
|  |  | **CG** | 1 | 154 | 158.2 |  |  |  | 2.7 | 2.3 | 3.2 |  |  |
|  |  | **GG** | 2 | 71 | 68.9 | 0.23 | *0.63* |  | 2.7 | 2.1 | 3.4 | 3.7 | *0.96* |
|  |  |  |  |  |  |  |  |  |  |  |  |  |  |
| ***GSTP1*** | **rs1138272**^d^ |  |  |  |  |  |  |  |  |  |  |  |  |
|  |  | **Missing** | 0 | 2 |  |  |  |  |  |  |  |  |  |
|  |  | **CC** | 0 | 277 | 273.7 |  |  |  | 2.7 | 2.4 | 3.0 |  |  |
|  |  | **CT** | 1 | 48 | 54.6 |  |  |  | 2.9 | 2.1 | 3.8 |  |  |
|  |  | **TT** | 2 | 6 | 2.7 | 4.79 | *0.03* |  | 4.4 | 1.9 | 10.2 | 64.5 | *0.49* |
|  |  | **CT + TT** |  | 54 |  |  |  |  | 3.0 | 2.3 | 4.0 | 11.8 | *0.47* |
|  |  |  |  |  |  |  |  |  |  |  |  |  |  |
| ***GSTP1*** | **rs749174** |  |  |  |  |  |  |  |  |  |  |  |  |
|  |  | **Missing** | 1 | 1 |  |  |  |  |  |  |  |  |  |
|  |  | **CC** | 0 | 146 | 145.8 |  |  |  | 2.6 | 2.2 | 3.1 |  |  |
|  |  | **CT** | 1 | 148 | 148.4 |  |  |  | 2.6 | 2.2 | 3.1 |  |  |
|  |  | **TT** | 2 | 38 | 37.8 | 0.00 | *0.96* |  | 3.1 | 2.2 | 4.4 | 18.6 | *0.65* |
|  |  |  |  |  |  |  |  |  |  |  |  |  |  |
| ***GSTP1*** | **rs1695** |  |  |  |  |  |  |  |  |  |  |  |  |
|  |  | **Missing** | 0 | 0 |  |  |  |  |  |  |  |  |  |
|  |  | **AA** | 0 | 150 | 149.3 |  |  |  | 2.6 | 2.2 | 3.0 |  |  |
|  |  | **AG** | 1 | 146 | 147.3 |  |  |  | 2.7 | 2.3 | 3.2 |  |  |
|  |  | **GG** | 2 | 37 | 36.3 | 0.03 | *0.87* |  | 3.2 | 2.3 | 4.6 | 26.3 | *0.49* |
|  |  |  |  |  |  |  |  |  |  |  |  |  |  |
| ***GSTP1*** | **rs762803** |  |  |  |  |  |  |  |  |  |  |  |  |
|  |  | **Missing** | 1 | 7 |  |  |  |  |  |  |  |  |  |
|  |  | **CC** | 0 | 109 | 106.7 |  |  |  | 2.7 | 2.2 | 3.3 |  |  |
|  |  | **CA** | 1 | 155 | 159.6 |  |  |  | 2.6 | 2.2 | 3.1 |  |  |
|  |  | **AA** | 2 | 62 | 59.7 | 0.27 | *0.60* |  | 2.9 | 2.2 | 3.8 | 6.2 | *0.77* |
|  |  |  |  |  |  |  |  |  |  |  |  |  |  |
| ***MnSOD*** | **rs5746151**^d^ |  |  |  |  |  |  |  |  |  |  |  |  |
|  |  | **Missing** | 0 | 0 |  |  |  |  |  |  |  |  |  |
|  |  | **GG** | 0 | 194 | 193.8 |  |  |  | 2.7 | 2.4 | 3.0 |  |  |
|  |  | **GA** | 1 | 37 | 37.4 |  |  |  | 2.6 | 1.8 | 3.7 |  |  |
|  |  | **AA** | 2 | 2 | 1.8 | 0.03 | *0.87* |  | 4.7 | 1.1 | 20.6 | 74.9 | *0.74* |
|  |  | **GA + AA** |  | 39 |  |  |  |  | 2.7 | 1.9 | 3.7 | -0.8 | *0.97* |
|  |  |  |  |  |  |  |  |  |  |  |  |  |  |
| ***MnSOD*** | **rs5746136** |  |  |  |  |  |  |  |  |  |  |  |  |
|  |  | **Missing** | 0 | 6 |  |  |  |  |  |  |  |  |  |
|  |  | **GG** | 0 | 160 | 157.6 |  |  |  | 2.9 | 2.5 | 3.4 |  |  |
|  |  | **GA** | 1 | 134 | 138.8 |  |  |  | 2.6 | 2.1 | 3.1 |  |  |
|  |  | **AA** | 2 | 33 | 30.6 | 0.40 | *0.53* |  | 2.3 | 1.6 | 3.3 | -22.1 | *0.36* |
|  |  |  |  |  |  |  |  |  |  |  |  |  |  |
| ***MnSOD*** | **rs4880** |  |  |  |  |  |  |  |  |  |  |  |  |
|  |  | **Missing** | 1 | 16 |  |  |  |  |  |  |  |  |  |
|  |  | **CC** | 0 | 71 | 83.3 |  |  |  | 3.4 | 2.7 | 4.3 |  |  |
|  |  | **CT** | 1 | 183 | 158.4 |  |  |  | 2.4 | 2.0 | 2.8 |  |  |
|  |  | **TT** | 2 | 63 | 75.3 | 7.65 | *0.01* |  | 2.7 | 2.1 | 3.5 | -19.8 | *0.04* |
|  |  |  |  |  |  |  |  |  |  |  |  |  |  |
| ***MnSOD*** | **rs6917589** |  |  |  |  |  |  |  |  |  |  |  |  |
|  |  | **Missing** | 0 | 6 |  |  |  |  |  |  |  |  |  |
|  |  | **TT** | 0 | 182 | 182.8 |  |  |  | 2.9 | 2.5 | 3.4 |  |  |
|  |  | **TC** | 1 | 125 | 123.4 |  |  |  | 2.5 | 2.1 | 3.0 |  |  |
|  |  | **CC** | 2 | 20 | 20.8 | 0.06 | *0.81* |  | 2.4 | 1.5 | 3.8 | -16.9 | *0.38* |
|  |  |  |  |  |  |  |  |  |  |  |  |  |  |
| ***MnSOD*** | **rs8031** |  |  |  |  |  |  |  |  |  |  |  |  |
|  |  | **Missing** | 1 | 7 |  |  |  |  |  |  |  |  |  |
|  |  | **TT** | 0 | 89 | 90.2 |  |  |  | 2.6 | 2.1 | 3.2 |  |  |
|  |  | **TA** | 1 | 165 | 162.6 |  |  |  | 2.6 | 2.2 | 3.0 |  |  |
|  |  | **AA** | 2 | 72 | 73.2 | 0.07 | *0.79* |  | 3.2 | 2.5 | 4.0 | 24.6 | *0.31* |
|  |  |  |  |  |  |  |  |  |  |  |  |  |  |
| ***MnSOD*** | **rs2842980**^d^ |  |  |  |  |  |  |  |  |  |  |  |  |
|  |  | **Missing** | 0 | 6 |  |  |  |  |  |  |  |  |  |
|  |  | **AA** | 0 | 203 | 209.1 |  |  |  | 2.7 | 2.3 | 3.1 |  |  |
|  |  | **AT** | 1 | 117 | 104.8 |  |  |  | 2.8 | 2.3 | 3.3 |  |  |
|  |  | **TT** | 2 | 7 | 13.1 | 4.46 | *0.03* |  | 1.5 | 0.7 | 3.2 | -46.0 | *0.29* |
|  |  | **AT + TT** |  | 124 |  |  |  |  | 2.7 | 2.2 | 3.2 | -1.7 | *0.88* |

Abbreviations: CL, confidence limit; Diff., difference; HWE, Hardy-Weinberg Equilibrium; LL, lower limit; MAP, Markers of Adenomatous Polyps; Prop. diff., proportional difference; SNP, single nucleotide polymorphism; UL, upper limit

^a^ Geometric means, 95% confidence limits, and *P*-values from general linear models, adjusted for sex (male/female) and body mass index (continuous)

^b^ Based on minor allele frequency in the European population in the 1000 Genomes Project Phase 3 (ensembl.org OR ncbi.nlm.nih.gov/projects/SNP)

^c^ Proportional difference, in percent, between mean plasma high sensitivity C-reactive protein concentration among those with a variant genotype relative to those with the common homozygous genotype; i.e.: ([variant mean - common mean] / common mean) x 100%

^d^ Heterozygous and/or variant homozygous genotypes with ≤ 10 participants were combined

**Supplemental Table 5.** Distributions of DNA base excision repair genotypes, and plasma high sensitivity C-reactive protein concentrations, according to the genotypes, in the pooled MAP I and MAP II cross-sectional studies (n = 333)^a^

| **Gene** | **SNP** | **Genotype** | **Weight** | **n** | **HWE** | | |  | **Geometric Mean** | | | **Prop. diff.**^c^ **(%)** | ***P*-value** |
| --- | --- | --- | --- | --- | --- | --- | --- | --- | --- | --- | --- | --- | --- |
|  |  |  |  |  | **Expected n**^b^ | **χ^2^** | ***P-value*** |  | **Mean** | **95% CL** | |  |  |
|  |  |  |  |  |  |  |  |  |  | **LL** | **UL** |  |  |
| ***APEX1*** | **rs3136814** |  |  |  |  |  |  |  |  |  |  |  |  |
|  |  | **Missing** | 0 | 3 |  |  |  |  |  |  |  |  |  |
|  |  | **AA** | 0 | 310 | 310.3 |  |  |  | 2.7 | 2.4 | 3.0 | - |  |
|  |  | **AC** | 1 | 20 | 19.4 | 0.02 | *0.89* |  | 3.1 | 1.9 | 4.9 | 14.0 | *0.59* |
|  |  |  |  |  |  |  |  |  |  |  |  |  |  |
| ***APEX1*** | **rs1130409** |  |  |  |  |  |  |  |  |  |  |  |  |
|  |  | **Missing** | 1 | 3 |  |  |  |  |  |  |  |  |  |
|  |  | **GG** | 0 | 85 | 86.0 |  |  |  | 2.7 | 2.1 | 3.4 | - |  |
|  |  | **GT** | 1 | 167 | 164.9 |  |  |  | 2.7 | 2.3 | 3.1 | -0.5 |  |
|  |  | **TT** | 2 | 78 | 79.0 | 0.05 | *0.82* |  | 2.7 | 2.1 | 3.4 | 0.0 | *1.00* |
|  |  |  |  |  |  |  |  |  |  |  |  |  |  |
| ***APEX1*** | **rs1760944** |  |  |  |  |  |  |  |  |  |  |  |  |
|  |  | **Missing** | 1 | 21 |  |  |  |  |  |  |  |  |  |
|  |  | **CC** | 0 | 112 | 112.7 |  |  |  | 2.9 | 2.4 | 3.6 | - |  |
|  |  | **CA** | 1 | 151 | 149.6 |  |  |  | 2.7 | 2.3 | 3.2 | -6.8 |  |
|  |  | **AA** | 2 | 49 | 49.7 | 0.03 | *0.87* |  | 2.2 | 1.6 | 3.0 | -23.6 | *0.34* |
|  |  |  |  |  |  |  |  |  |  |  |  |  |  |
| ***FEN1*** | **rs412334**^d^ |  |  |  |  |  |  |  |  |  |  |  |  |
|  |  | **Missing** | 0 | 14 |  |  |  |  |  |  |  |  |  |
|  |  | **GG** | 0 | 221 | 226.1 |  |  |  | 2.7 | 2.4 | 3.2 | - |  |
|  |  | **GA** | 1 | 96 | 85.7 |  |  |  | 2.8 | 2.3 | 3.5 | 2.3 |  |
|  |  | **AA** | 2 | 3 | 8.1 | 4.58 | *0.03* |  | 1.5 | 0.5 | 5.2 | -43.8 | *0.63* |
|  |  | **GA+AA** |  | 99 |  |  |  |  | 2.8 | 2.2 | 3.4 | 0.4 | *0.97* |
|  |  |  |  |  |  |  |  |  |  |  |  |  |  |
| ***LIG1*** | **rs419664** |  |  |  |  |  |  |  |  |  |  |  |  |
|  |  | **Missing** | 1 | 2 |  |  |  |  |  |  |  |  |  |
|  |  | **GG** | 0 | 109 | 105.1 |  |  |  | 2.7 | 2.2 | 3.3 | - |  |
|  |  | **GT** | 1 | 155 | 162.8 |  |  |  | 2.6 | 2.2 | 3.0 | -4.4 |  |
|  |  | **TT** | 2 | 67 | 63.1 | 0.77 | *0.38* |  | 2.4 | 1.9 | 3.1 | -10.7 | *0.73* |
|  |  |  |  |  |  |  |  |  |  |  |  |  |  |
| ***LIG1*** | **rs156641** |  |  |  |  |  |  |  |  |  |  |  |  |
|  |  | **Missing** | 1 | 4 |  |  |  |  |  |  |  |  |  |
|  |  | **GG** | 0 | 129 | 123.4 |  |  |  | 2.6 | 2.2 | 3.1 | - |  |
|  |  | **GA** | 1 | 145 | 156.2 |  |  |  | 2.7 | 2.3 | 3.2 | 4.8 |  |
|  |  | **AA** | 2 | 55 | 49.4 | 1.69 | *0.19* |  | 3.0 | 2.3 | 4.0 | 15.3 | *0.71* |
|  |  |  |  |  |  |  |  |  |  |  |  |  |  |
| ***LIG1*** | **rs2288881**^d^ |  |  |  |  |  |  |  |  |  |  |  |  |
|  |  | **Missing** | 0 | 3 |  |  |  |  |  |  |  |  |  |
|  |  | **GG** | 0 | 301 | 299.7 |  |  |  | 2.7 | 2.4 | 3.1 | - |  |
|  |  | **GA** | 1 | 27 | 29.5 |  |  |  | 2.2 | 1.5 | 3.3 | -19.1 |  |
|  |  | **AA** | 2 | 2 | 0.7 | 2.45 | *0.12* |  | 1.5 | 0.3 | 6.7 | -45.2 | *0.46* |
|  |  | **GA+AA** |  | 29 |  |  |  |  | 2.2 | 1.5 | 3.2 | -21.2 | *0.25* |
|  |  |  |  |  |  |  |  |  |  |  |  |  |  |
| ***LIG1*** | **rs3730947** |  |  |  |  |  |  |  |  |  |  |  |  |
|  |  | **Missing** | 0 | 1 |  |  |  |  |  |  |  |  |  |
|  |  | **GG** | 0 | 332 |  |  |  |  | 2.7 | 2.4 | 3.0 |  | *NA* |
|  |  |  |  |  |  |  |  |  |  |  |  |  |  |
| ***LIG1*** | **rs3731037**^d^ |  |  |  |  |  |  |  |  |  |  |  |  |
|  |  | **Missing** | 0 | 2 |  |  |  |  |  |  |  |  |  |
|  |  | **CC** | 0 | 260 | 256.7 |  |  |  | 2.6 | 2.3 | 2.9 | - |  |
|  |  | **CT** | 1 | 63 | 69.6 |  |  |  | 3.3 | 2.5 | 4.2 | 25.7 |  |
|  |  | **TT** | 2 | 8 | 4.7 | 2.95 | *0.09* |  | 1.6 | 0.8 | 3.3 | -39.4 | *0.11* |
|  |  | **CT+TT** |  | 71 |  |  |  |  | 3.0 | 2.4 | 3.8 | 15.9 | *0.30* |
|  |  |  |  |  |  |  |  |  |  |  |  |  |  |
| ***LIG1*** | **rs411073** |  |  |  |  |  |  |  |  |  |  |  |  |
|  |  | **Missing** | 1 | 7 |  |  |  |  |  |  |  |  |  |
|  |  | **CC** | 0 | 107 | 102.2 |  |  |  | 3.0 | 2.4 | 3.6 | - |  |
|  |  | **CT** | 1 | 151 | 160.7 |  |  |  | 2.6 | 2.2 | 3.0 | -13.2 |  |
|  |  | **TT** | 2 | 68 | 63.2 | 1.18 | *0.28* |  | 2.7 | 2.1 | 3.5 | -9.2 | *0.57* |
|  |  |  |  |  |  |  |  |  |  |  |  |  |  |
| ***LIG1*** | **rs3730908**^d^ |  |  |  |  |  |  |  |  |  |  |  |  |
|  |  | **Missing** | 0 | 2 |  |  |  |  |  |  |  |  |  |
|  |  | **CC** | 0 | 307 | 306.5 |  |  |  | 2.7 | 2.4 | 3.1 | - |  |
|  |  | **CT** | 1 | 23 | 24.1 |  |  |  | 2.2 | 1.4 | 3.4 | -19.6 |  |
|  |  | **TT** | 2 | 1 | 0.5 | 0.64 | *0.42* |  | 1.4 | 0.2 | 11.4 | -48.5 | *0.53* |
|  |  | **CT+TT** |  | 24 |  |  |  |  | 2.2 | 1.4 | 3.3 | -21.2 | *0.29* |
|  |  |  |  |  |  |  |  |  |  |  |  |  |  |
| ***LIG1*** | **rs20579**^d^ |  |  |  |  |  |  |  |  |  |  |  |  |
|  |  | **Missing** | 0 | 1 |  |  |  |  |  |  |  |  |  |
|  |  | **CC** | 0 | 251 | 251.6 |  |  |  | 2.6 | 2.3 | 3.0 | - |  |
|  |  | **CT** | 1 | 76 | 74.9 |  |  |  | 2.9 | 2.3 | 3.7 | 9.2 |  |
|  |  | **TT** | 2 | 5 | 5.6 | 0.08 | *0.78* |  | 2.1 | 0.8 | 5.3 | -21.4 | *0.70* |
|  |  | **CT+TT** |  | 82 |  |  |  |  | 2.8 | 2.2 | 3.6 | 7.0 | *0.62* |
|  |  |  |  |  |  |  |  |  |  |  |  |  |  |
| ***LIG1*** | **rs3730881** |  |  |  |  |  |  |  |  |  |  |  |  |
|  |  | **Missing** | 0 | 0 |  |  |  |  |  |  |  |  |  |
|  |  | **CC** | 0 | 327 | 327.0 |  |  |  | 2.7 | 2.4 | 3.0 | - |  |
|  |  | **CT** | 1 | 6 | 5.9 | 0.00 | *0.98* |  | 2.1 | 0.9 | 5.0 | -21.1 | *0.59* |
|  |  |  |  |  |  |  |  |  |  |  |  |  |  |
| ***LIG1*** | **rs3730914**^d^ |  |  |  |  |  |  |  |  |  |  |  |  |
|  |  | **Missing** | 0 | 5 |  |  |  |  |  |  |  |  |  |
|  |  | **CC** | 0 | 227 | 228.1 |  |  |  | 2.6 | 2.3 | 3.0 | - |  |
|  |  | **CT** | 1 | 93 | 90.9 |  |  |  | 3.1 | 2.5 | 3.8 | 16.8 |  |
|  |  | **TT** | 2 | 8 | 9.1 | 0.18 | *0.67* |  | 1.4 | 0.7 | 2.9 | -48.1 | *0.09* |
|  |  | **CT+TT** |  | 101 |  |  |  |  | 2.9 | 2.3 | 3.6 | 9.8 | *0.46* |
|  |  |  |  |  |  |  |  |  |  |  |  |  |  |
| ***LIG1*** | **rs3731003** |  |  |  |  |  |  |  |  |  |  |  |  |
|  |  | **Missing** | 0 | 3 |  |  |  |  |  |  |  |  |  |
|  |  | **CC** | 0 | 330 |  |  |  |  | 2.7 | 2.4 | 3.0 |  | *NA* |
|  |  |  |  |  |  |  |  |  |  |  |  |  |  |
| ***LIG1*** | **rs3730837**^d^ |  |  |  |  |  |  |  |  |  |  |  |  |
|  |  | **Missing** | 0 | 11 |  |  |  |  |  |  |  |  |  |
|  |  | **AA** | 0 | 251 | 252.3 |  |  |  | 2.5 | 2.2 | 2.8 | - |  |
|  |  | **AG** | 1 | 68 | 65.5 |  |  |  | 3.0 | 2.3 | 3.8 | 21.0 |  |
|  |  | **GG** | 2 | 3 | 4.3 | 0.47 | *0.49* |  | 2.0 | 0.3 | 14.1 | -20.4 | *0.45* |
|  |  | **AG+GG** |  | 71 |  |  |  |  | 3.0 | 2.3 | 3.8 | 20.3 | *0.21* |
|  |  |  |  |  |  |  |  |  |  |  |  |  |  |
| ***LIG1*** | **rs274862** |  |  |  |  |  |  |  |  |  |  |  |  |
|  |  | **Missing** | 1 | 8 |  |  |  |  |  |  |  |  |  |
|  |  | **TT** | 0 | 117 | 115.2 |  |  |  | 2.6 | 2.2 | 3.2 | - |  |
|  |  | **TC** | 1 | 153 | 156.6 |  |  |  | 2.7 | 2.3 | 3.2 | 2.6 |  |
|  |  | **CC** | 2 | 55 | 53.2 | 0.17 | *0.68* |  | 3.0 | 2.2 | 3.9 | 13.7 | *0.75* |
|  |  |  |  |  |  |  |  |  |  |  |  |  |  |
| ***LIG1*** | **rs3730912**^d^ |  |  |  |  |  |  |  |  |  |  |  |  |
|  |  | **Missing** | 0 | 1 |  |  |  |  |  |  |  |  |  |
|  |  | **CC** | 0 | 257 | 254.2 |  |  |  | 2.6 | 2.3 | 2.9 | - |  |
|  |  | **CA** | 1 | 67 | 72.6 |  |  |  | 3.4 | 2.6 | 4.4 | 31.8 |  |
|  |  | **AA** | 2 | 8 | 5.2 | 1.99 | *0.16* |  | 1.6 | 0.8 | 3.3 | -38.8 | *0.06* |
|  |  | **CA+AA** |  | 75 |  |  |  |  | 3.1 | 2.5 | 4.0 | 21.6 | *0.16* |
|  |  |  |  |  |  |  |  |  |  |  |  |  |  |
| ***LIG1*** | **rs20580** |  |  |  |  |  |  |  |  |  |  |  |  |
|  |  | **Missing** | 1 | 8 |  |  |  |  |  |  |  |  |  |
|  |  | **CC** | 0 | 84 | 76.3 |  |  |  | 2.4 | 1.9 | 3.1 | - |  |
|  |  | **CA** | 1 | 147 | 162.3 |  |  |  | 2.7 | 2.2 | 3.2 | 8.3 |  |
|  |  | **AA** | 2 | 94 | 86.3 | 2.90 | *0.09* |  | 3.0 | 2.4 | 3.7 | 22.5 | *0.44* |
|  |  |  |  |  |  |  |  |  |  |  |  |  |  |
| ***LIG3*** | **rs3135974**^d^ |  |  |  |  |  |  |  |  |  |  |  |  |
|  |  | **Missing** | 0 | 1 |  |  |  |  |  |  |  |  |  |
|  |  | **GG** | 0 | 273 | 272.9 |  |  |  | 2.6 | 2.3 | 3.0 | - |  |
|  |  | **GA** | 1 | 56 | 56.2 |  |  |  | 3.0 | 2.2 | 3.9 | 12.6 |  |
|  |  | **AA** | 2 | 3 | 2.9 | 0.00 | *0.95* |  | 4.8 | 1.4 | 15.9 | 82.9 | *0.48* |
|  |  | **GA+AA** |  | 59 |  |  |  |  | 3.0 | 2.3 | 4.0 | 15.4 | *0.35* |
|  |  |  |  |  |  |  |  |  |  |  |  |  |  |
| ***LIG3*** | **rs3135998** |  |  |  |  |  |  |  |  |  |  |  |  |
|  |  | **Missing** | 1 | 0 |  |  |  |  |  |  |  |  |  |
|  |  | **GG** | 0 | 112 | 109.0 |  |  |  | 2.9 | 2.3 | 3.5 | - |  |
|  |  | **GA** | 1 | 157 | 163.0 |  |  |  | 2.5 | 2.1 | 3.0 | -12.0 |  |
|  |  | **AA** | 2 | 64 | 61.0 | 0.46 | *0.50* |  | 2.9 | 2.2 | 3.7 | 0.8 | *0.53* |
|  |  |  |  |  |  |  |  |  |  |  |  |  |  |
| ***LIG3*** | **rs3135989**^d^ |  |  |  |  |  |  |  |  |  |  |  |  |
|  |  | **Missing** | 0 | 2 |  |  |  |  |  |  |  |  |  |
|  |  | **TT** | 0 | 295 | 295.0 |  |  |  | 2.7 | 2.4 | 3.0 | - |  |
|  |  | **TG** | 1 | 35 | 34.9 |  |  |  | 2.6 | 1.8 | 3.7 | -2.0 |  |
|  |  | **GG** | 2 | 1 | 1.0 | 0.00 | *0.97* |  | 5.4 | 0.7 | 44.0 | 102.0 | *0.80* |
|  |  | **TG+GG** |  | 36 |  |  |  |  | 2.7 | 1.9 | 3.8 | 0.0 | *1.00* |
|  |  |  |  |  |  |  |  |  |  |  |  |  |  |
| ***LIG3*** | **rs3135967** |  |  |  |  |  |  |  |  |  |  |  |  |
|  |  | **Missing** | 1 | 5 |  |  |  |  |  |  |  |  |  |
|  |  | **AA** | 0 | 102 | 101.5 |  |  |  | 3.0 | 2.5 | 3.7 | - |  |
|  |  | **AG** | 1 | 161 | 161.9 |  |  |  | 2.5 | 2.1 | 3.0 | -17.2 |  |
|  |  | **GG** | 2 | 65 | 64.5 | 0.01 | *0.92* |  | 2.6 | 2.0 | 3.4 | -14.5 | *0.36* |
|  |  |  |  |  |  |  |  |  |  |  |  |  |  |
| ***LIG3*** | **rs2074516**^d^ |  |  |  |  |  |  |  |  |  |  |  |  |
|  |  | **Missing** | 0 | 1 |  |  |  |  |  |  |  |  |  |
|  |  | **GG** | 0 | 270 | 270.2 |  |  |  | 2.6 | 2.3 | 3.0 | - |  |
|  |  | **GC** | 1 | 59 | 58.6 |  |  |  | 3.1 | 2.4 | 4.0 | 18.8 |  |
|  |  | **CC** | 2 | 3 | 3.2 | 0.01 | *0.91* |  | 4.8 | 1.4 | 15.9 | 84.6 | *0.34* |
|  |  | **GC+CC** |  | 62 |  |  |  |  | 3.2 | 2.4 | 4.1 | 21.4 | *0.19* |
|  |  |  |  |  |  |  |  |  |  |  |  |  |  |
| ***MBD4*** | **rs3138360**^d^ |  |  |  |  |  |  |  |  |  |  |  |  |
|  |  | **Missing** | 0 | 1 |  |  |  |  |  |  |  |  |  |
|  |  | **GG** | 0 | 297 | 297.0 |  |  |  | 2.7 | 2.4 | 3.1 | - |  |
|  |  | **GA** | 1 | 34 | 34.0 |  |  |  | 2.4 | 1.7 | 3.4 | -13.5 |  |
|  |  | **AA** | 2 | 1 | 1.0 | 0.00 | *0.98* |  | 2.2 | 0.3 | 17.7 | -19.8 | *0.74* |
|  |  | **GA+AA** |  | 35 |  |  |  |  | 2.4 | 1.7 | 3.4 | -13.6 | *0.44* |
|  |  |  |  |  |  |  |  |  |  |  |  |  |  |
| ***MBD4*** | **rs10342**^d^ |  |  |  |  |  |  |  |  |  |  |  |  |
|  |  | **Missing** | 0 | 1 |  |  |  |  |  |  |  |  |  |
|  |  | **GG** | 0 | 275 | 272.9 |  |  |  | 2.7 | 2.4 | 3.1 | - |  |
|  |  | **GA** | 1 | 52 | 56.2 |  |  |  | 2.7 | 2.0 | 3.6 | -1.1 |  |
|  |  | **AA** | 2 | 5 | 2.9 | 1.86 | *0.17* |  | 2.2 | 0.9 | 5.5 | -20.4 | *0.89* |
|  |  | **GA+AA** |  | 57 |  |  |  |  | 2.6 | 2.0 | 3.5 | -3.0 | *0.84* |
|  |  |  |  |  |  |  |  |  |  |  |  |  |  |
| ***MBD4*** | **rs2005618**^d^ |  |  |  |  |  |  |  |  |  |  |  |  |
|  |  | **Missing** | 0 | 0 |  |  |  |  |  |  |  |  |  |
|  |  | **TT** | 0 | 266 | 264.9 |  |  |  | 2.7 | 2.4 | 3.1 | - |  |
|  |  | **TC** | 1 | 62 | 64.2 |  |  |  | 2.6 | 2.0 | 3.4 | -6.0 |  |
|  |  | **CC** | 2 | 5 | 3.9 | 0.40 | *0.53* |  | 2.2 | 0.9 | 5.5 | -20.7 | *0.83* |
|  |  | **TC+CC** |  | 67 |  |  |  |  | 2.5 | 2.0 | 3.3 | -7.2 | *0.61* |
|  |  |  |  |  |  |  |  |  |  |  |  |  |  |
| ***MBD4*** | **rs2311394**^d^ |  |  |  |  |  |  |  |  |  |  |  |  |
|  |  | **Missing** | 0 | 6 |  |  |  |  |  |  |  |  |  |
|  |  | **TT** | 0 | 267 | 266.1 |  |  |  | 2.8 | 2.4 | 3.1 | - |  |
|  |  | **TC** | 1 | 56 | 57.7 |  |  |  | 2.6 | 2.0 | 3.4 | -5.6 |  |
|  |  | **CC** | 2 | 4 | 3.1 | 0.30 | *0.59* |  | 2.2 | 0.8 | 6.2 | -19.9 | *0.86* |
|  |  | **TC+CC** |  | 60 |  |  |  |  | 2.6 | 2.0 | 3.4 | -6.6 | *0.65* |
|  |  |  |  |  |  |  |  |  |  |  |  |  |  |
| ***MBD4*** | **rs2307293** |  |  |  |  |  |  |  |  |  |  |  |  |
|  |  | **Missing** | 0 | 4 |  |  |  |  |  |  |  |  |  |
|  |  | **GG** | 0 | 326 | 326.0 |  |  |  | 2.7 | 2.4 | 3.1 | - |  |
|  |  | **GC** | 1 | 3 | 3.0 | 0.00 | *0.99* |  | 1.0 | 0.3 | 3.2 | -65.0 | *0.09* |
|  |  |  |  |  |  |  |  |  |  |  |  |  |  |
| ***MBD4*** | **rs3138326**^d^ |  |  |  |  |  |  |  |  |  |  |  |  |
|  |  | **Missing** | 0 | 14 |  |  |  |  |  |  |  |  |  |
|  |  | **AA** | 0 | 258 | 256.4 |  |  |  | 2.7 | 2.4 | 3.1 | - |  |
|  |  | **AT** | 1 | 56 | 59.2 |  |  |  | 2.7 | 2.0 | 3.5 | -2.0 |  |
|  |  | **TT** | 2 | 5 | 3.4 | 0.92 | *0.34* |  | 2.2 | 0.9 | 5.5 | -20.1 | *0.89* |
|  |  | **AT+TT** |  | 61 |  |  |  |  | 2.6 | 2.0 | 3.4 | -3.6 | *0.81* |
|  |  |  |  |  |  |  |  |  |  |  |  |  |  |
| ***MPG*** | **rs3176415** |  |  |  |  |  |  |  |  |  |  |  |  |
|  |  | **Missing** | 1 | 8 |  |  |  |  |  |  |  |  |  |
|  |  | **GG** | 0 | 100 | 101.4 |  |  |  | 2.6 | 2.1 | 3.1 | - |  |
|  |  | **GA** | 1 | 163 | 160.3 |  |  |  | 2.8 | 2.3 | 3.2 | 7.6 |  |
|  |  | **AA** | 2 | 62 | 63.4 | 0.09 | *0.76* |  | 2.7 | 2.1 | 3.5 | 6.2 | *0.86* |
|  |  |  |  |  |  |  |  |  |  |  |  |  |  |
| ***MPG*** | **rs2541622**^d^ |  |  |  |  |  |  |  |  |  |  |  |  |
|  |  | **Missing** | 0 | 4 |  |  |  |  |  |  |  |  |  |
|  |  | **CC** | 0 | 223 | 224.0 |  |  |  | 2.8 | 2.5 | 3.2 | - |  |
|  |  | **CT** | 1 | 97 | 94.9 |  |  |  | 2.5 | 2.0 | 3.1 | -11.9 |  |
|  |  | **TT** | 2 | 9 | 10.0 | 0.16 | *0.69* |  | 2.3 | 1.2 | 4.7 | -17.5 | *0.56* |
|  |  | **CT+TT** |  | 106 |  |  |  |  | 2.5 | 2.0 | 3.0 | -12.4 | *0.29* |
|  |  |  |  |  |  |  |  |  |  |  |  |  |  |
| ***MPG*** | **rs3176424** |  |  |  |  |  |  |  |  |  |  |  |  |
|  |  | **Missing** | 0 | 2 |  |  |  |  |  |  |  |  |  |
|  |  | **AA** | 0 | 323 | 323.0 |  |  |  | 2.7 | 2.4 | 3.0 | - |  |
|  |  | **AG** | 1 | 8 | 7.9 | 0.00 | *0.97* |  | 3.5 | 1.7 | 7.3 | 30.6 | *0.48* |
|  |  |  |  |  |  |  |  |  |  |  |  |  |  |
| ***MUTYH*** | **rs3219476** |  |  |  |  |  |  |  |  |  |  |  |  |
|  |  | **Missing** | 1 | 0 |  |  |  |  |  |  |  |  |  |
|  |  | **GG** | 0 | 134 | 137.5 |  |  |  | 2.6 | 2.1 | 3.1 | - |  |
|  |  | **GT** | 1 | 160 | 152.9 |  |  |  | 2.8 | 2.4 | 3.3 | 9.1 |  |
|  |  | **TT** | 2 | 39 | 42.5 | 0.71 | *0.40* |  | 2.9 | 2.0 | 4.0 | 11.7 | *0.74* |
|  |  |  |  |  |  |  |  |  |  |  |  |  |  |
| ***MUTYH*** | **rs3219484**^d^ |  |  |  |  |  |  |  |  |  |  |  |  |
|  |  | **Missing** | 0 | 1 |  |  |  |  |  |  |  |  |  |
|  |  | **GG** | 0 | 285 | 285.7 |  |  |  | 2.8 | 2.5 | 3.2 | - |  |
|  |  | **GA** | 1 | 46 | 44.5 |  |  |  | 2.1 | 1.5 | 2.8 | -26.3 |  |
|  |  | **AA** | 2 | 1 | 1.7 | 0.36 | *0.55* |  | 0.6 | 0.1 | 4.7 | -78.8 | *0.070* |
|  |  | **GA+AA** |  | 47 |  |  |  |  | 2.0 | 1.5 | 2.7 | -28.2 | *0.05* |
|  |  |  |  |  |  |  |  |  |  |  |  |  |  |
| ***MUTYH*** | **rs3219494** |  |  |  |  |  |  |  |  |  |  |  |  |
|  |  | **Missing** | 0 | 0 |  |  |  |  |  |  |  |  |  |
|  |  | **GG** | 0 | 333 |  |  |  |  | 2.7 | 2.4 | 3.0 | - | *NA* |
|  |  |  |  |  |  |  |  |  |  |  |  |  |  |
| ***MUTYH*** | **rs3219463** |  |  |  |  |  |  |  |  |  |  |  |  |
|  |  | **Missing** | 0 | 3 |  |  |  |  |  |  |  |  |  |
|  |  | **GG** | 0 | 179 | 184.9 |  |  |  | 2.7 | 2.4 | 3.2 | - |  |
|  |  | **GA** | 1 | 136 | 124.2 |  |  |  | 2.7 | 2.2 | 3.2 | -2.5 |  |
|  |  | **AA** | 2 | 15 | 20.9 | 2.95 | *0.09* |  | 3.1 | 1.8 | 5.2 | 11.6 | *0.89* |
|  |  |  |  |  |  |  |  |  |  |  |  |  |  |
| ***MUTYH*** | **rs3219489** |  |  |  |  |  |  |  |  |  |  |  |  |
|  |  | **Missing** | 0 | 0 |  |  |  |  |  |  |  |  |  |
|  |  | **GG** | 0 | 180 | 184.7 |  |  |  | 2.7 | 2.3 | 3.2 | - |  |
|  |  | **GC** | 1 | 136 | 126.6 |  |  |  | 2.6 | 2.2 | 3.2 | -2.2 |  |
|  |  | **CC** | 2 | 17 | 21.7 | 1.83 | *0.18* |  | 3.1 | 1.9 | 5.2 | 15.5 | *0.83* |
|  |  |  |  |  |  |  |  |  |  |  |  |  |  |
| ***MUTYH*** | **rs3219493**^d^ |  |  |  |  |  |  |  |  |  |  |  |  |
|  |  | **Missing** | 0 | 8 |  |  |  |  |  |  |  |  |  |
|  |  | **GG** | 0 | 161 | 163.8 |  |  |  | 2.6 | 2.3 | 3.0 | - |  |
|  |  | **GC** | 1 | 62 | 56.3 |  |  |  | 2.8 | 2.1 | 3.6 | 5.7 |  |
|  |  | **CC** | 2 | 2 | 4.8 | 2.29 | *0.13* |  | 8.8 | 2.0 | 38.7 | 237.1 | *0.26* |
|  |  | **GC+CC** |  | 64 |  |  |  |  | 2.9 | 2.2 | 3.7 | 9.5 | *0.54* |
|  |  |  |  |  |  |  |  |  |  |  |  |  |  |
| ***OGG1*** | **rs125701**^d^ |  |  |  |  |  |  |  |  |  |  |  |  |
|  |  | **Missing** | 0 | 6 |  |  |  |  |  |  |  |  |  |
|  |  | **GG** | 0 | 235 | 233.0 |  |  |  | 2.7 | 2.3 | 3.1 | - |  |
|  |  | **GA** | 1 | 82 | 86.1 |  |  |  | 2.7 | 2.2 | 3.4 | 2.2 |  |
|  |  | **AA** | 2 | 10 | 8.0 | 0.74 | *0.39* |  | 2.9 | 1.5 | 5.5 | 6.6 | *0.97* |
|  |  | **GA+AA** |  | 92 |  |  |  |  | 2.7 | 2.2 | 3.4 | 2.6 | *0.84* |
|  |  |  |  |  |  |  |  |  |  |  |  |  |  |
| ***OGG1*** | **rs1805373** |  |  |  |  |  |  |  |  |  |  |  |  |
|  |  | **Missing** | 0 | 0 |  |  |  |  |  |  |  |  |  |
|  |  | **GG** | 0 | 333 |  |  |  |  | 2.7 | 2.4 | 3.0 | - | *NA* |
|  |  |  |  |  |  |  |  |  |  |  |  |  |  |
| ***OGG1*** | **rs2072668** |  |  |  |  |  |  |  |  |  |  |  |  |
|  |  | **Missing** | 0 | 1 |  |  |  |  |  |  |  |  |  |
|  |  | **CC** | 0 | 188 | 187.5 |  |  |  | 2.8 | 2.4 | 3.2 | - |  |
|  |  | **CG** | 1 | 123 | 124.0 |  |  |  | 2.6 | 2.2 | 3.2 | -4.5 |  |
|  |  | **GG** | 2 | 21 | 20.5 | 0.02 | *0.88* |  | 2.5 | 1.6 | 3.9 | -10.9 | *0.86* |
|  |  |  |  |  |  |  |  |  |  |  |  |  |  |
| ***OGG1*** | **rs3219008** |  |  |  |  |  |  |  |  |  |  |  |  |
|  |  | **Missing** | 0 | 4 |  |  |  |  |  |  |  |  |  |
|  |  | **AA** | 0 | 193 | 186.9 |  |  |  | 2.7 | 2.3 | 3.1 | - |  |
|  |  | **AG** | 1 | 110 | 122.1 |  |  |  | 2.7 | 2.2 | 3.3 | -1.3 |  |
|  |  | **GG** | 2 | 26 | 19.9 | 3.24 | *0.07* |  | 2.7 | 1.8 | 4.1 | 1.2 | *0.99* |
|  |  |  |  |  |  |  |  |  |  |  |  |  |  |
| ***OGG1*** | **rs159153** |  |  |  |  |  |  |  |  |  |  |  |  |
|  |  | **Missing** | 0 | 7 |  |  |  |  |  |  |  |  |  |
|  |  | **TT** | 0 | 167 | 158.1 |  |  |  | 2.8 | 2.4 | 3.3 | - |  |
|  |  | **TC** | 1 | 120 | 137.9 |  |  |  | 2.5 | 2.1 | 3.0 | -11.6 |  |
|  |  | **CC** | 2 | 39 | 30.1 | 5.48 | *0.02* |  | 2.9 | 2.0 | 4.0 | 1.7 | *0.85* |
|  |  |  |  |  |  |  |  |  |  |  |  |  |  |
| ***OGG1*** | **rs293795** |  |  |  |  |  |  |  |  |  |  |  |  |
|  |  | **Missing** | 0 | 0 |  |  |  |  |  |  |  |  |  |
|  |  | **TT** | 0 | 224 | 220.5 |  |  |  | 2.7 | 2.3 | 3.1 | - |  |
|  |  | **TC** | 1 | 94 | 100.9 |  |  |  | 2.8 | 2.3 | 3.5 | 5.5 |  |
|  |  | **CC** | 2 | 15 | 11.5 | 1.56 | *0.21* |  | 2.4 | 1.4 | 4.2 | -8.4 | *0.86* |
|  |  |  |  |  |  |  |  |  |  |  |  |  |  |
| ***PNKP*** | **rs3739206** |  |  |  |  |  |  |  |  |  |  |  |  |
|  |  | **Missing** | 0 | 1 |  |  |  |  |  |  |  |  |  |
|  |  | **TT** | 0 | 331 | 330.0 |  |  |  | 2.7 | 2.4 | 3.0 | - |  |
|  |  | **GG** | 2 | 1 | 0.0 | 330.01 | *< 0.001* |  | 2.0 | 0.3 | 16.3 | -24.6 | *0.79* |
|  |  |  |  |  |  |  |  |  |  |  |  |  |  |
| ***PNKP*** | **rs2257103** |  |  |  |  |  |  |  |  |  |  |  |  |
|  |  | **Missing** | 1 | 7 |  |  |  |  |  |  |  |  |  |
|  |  | **CC** | 0 | 127 | 119.0 |  |  |  | 2.9 | 2.4 | 3.4 | - |  |
|  |  | **CT** | 1 | 140 | 155.9 |  |  |  | 2.6 | 2.1 | 3.1 | -10.6 |  |
|  |  | **TT** | 2 | 59 | 51.0 | 3.39 | *0.07* |  | 2.7 | 2.1 | 3.5 | -6.1 | *0.69* |
|  |  |  |  |  |  |  |  |  |  |  |  |  |  |
| ***PNKP*** | **rs3739186** |  |  |  |  |  |  |  |  |  |  |  |  |
|  |  | **Missing** | 0 | 1 |  |  |  |  |  |  |  |  |  |
|  |  | **TT** | 0 | 332 |  |  |  |  | 2.7 | 2.4 | 3.0 | - | *NA* |
|  |  |  |  |  |  |  |  |  |  |  |  |  |  |
| ***POLB*** | **rs2979896**^d^ |  |  |  |  |  |  |  |  |  |  |  |  |
|  |  | **Missing** | 0 | 1 |  |  |  |  |  |  |  |  |  |
|  |  | **TT** | 0 | 292 | 293.2 |  |  |  | 2.7 | 2.4 | 3.1 | - |  |
|  |  | **TG** | 1 | 40 | 37.6 | 0.16 | *0.69* |  | 2.5 | 1.8 | 3.4 | -9.1 | *0.59* |
|  |  |  |  |  |  |  |  |  |  |  |  |  |  |
| ***POLB*** | **rs3136811**^d^ |  |  |  |  |  |  |  |  |  |  |  |  |
|  |  | **Missing** | 0 | 1 |  |  |  |  |  |  |  |  |  |
|  |  | **CC** | 0 | 291 | 292.3 |  |  |  | 2.7 | 2.4 | 3.1 | - |  |
|  |  | **CG** | 1 | 41 | 38.5 | 0.17 | *0.68* |  | 2.5 | 1.8 | 3.5 | -7.1 | *0.68* |
|  |  |  |  |  |  |  |  |  |  |  |  |  |  |
| ***POLB*** | **rs3136797** |  |  |  |  |  |  |  |  |  |  |  |  |
|  |  | **Missing** | 0 | 9 |  |  |  |  |  |  |  |  |  |
|  |  | **CC** | 0 | 316 | 316.0 |  |  |  | 2.7 | 2.4 | 3.0 | - |  |
|  |  | **CG** | 1 | 8 | 7.9 | 0.00 | *0.97* |  | 3.0 | 1.4 | 6.3 | 12.6 | *0.76* |
|  |  |  |  |  |  |  |  |  |  |  |  |  |  |
| ***SMUG1*** | **rs2233920** |  |  |  |  |  |  |  |  |  |  |  |  |
|  |  | **Missing** | 0 | 1 |  |  |  |  |  |  |  |  |  |
|  |  | **GG** | 0 | 332 |  |  |  |  | 2.7 | 2.4 | 3.0 | - | *NA* |
|  |  |  |  |  |  |  |  |  |  |  |  |  |  |
| ***SMUG1*** | **rs3136386**^d^ |  |  |  |  |  |  |  |  |  |  |  |  |
|  |  | **Missing** | 0 | 2 |  |  |  |  |  |  |  |  |  |
|  |  | **CC** | 0 | 305 | 304.6 |  |  |  | 2.7 | 2.4 | 3.0 | - |  |
|  |  | **CG** | 1 | 25 | 25.9 |  |  |  | 2.9 | 1.9 | 4.4 | 8.3 |  |
|  |  | **GG** | 2 | 1 | 0.6 | 0.40 | *0.53* |  | 1.3 | 0.2 | 10.3 | -51.1 | *0.74* |
|  |  | **CG+GG** |  | 26 |  |  |  |  | 2.8 | 1.9 | 4.2 | 5.1 | *0.82* |
|  |  |  |  |  |  |  |  |  |  |  |  |  |  |
| ***SMUG1*** | **rs971** |  |  |  |  |  |  |  |  |  |  |  |  |
|  |  | **Missing** | 0 | 2 |  |  |  |  |  |  |  |  |  |
|  |  | **CC** | 0 | 143 | 137.1 |  |  |  | 2.4 | 2.0 | 2.8 | - |  |
|  |  | **CT** | 1 | 140 | 151.9 |  |  |  | 2.9 | 2.5 | 3.5 | 25.0 |  |
|  |  | **TT** | 2 | 48 | 42.1 | 2.02 | *0.16* |  | 3.1 | 2.3 | 4.2 | 32.6 | *0.12* |
|  |  |  |  |  |  |  |  |  |  |  |  |  |  |
| ***SMUG1*** | **rs2279402** |  |  |  |  |  |  |  |  |  |  |  |  |
|  |  | **Missing** | 1 | 1 |  |  |  |  |  |  |  |  |  |
|  |  | **CC** | 0 | 104 | 98.7 |  |  |  | 2.4 | 2.0 | 3.0 | - |  |
|  |  | **CT** | 1 | 154 | 164.6 |  |  |  | 2.8 | 2.3 | 3.3 | 13.6 |  |
|  |  | **TT** | 2 | 74 | 68.7 | 1.39 | *0.24* |  | 3.0 | 2.4 | 3.8 | 23.3 | *0.41* |
|  |  |  |  |  |  |  |  |  |  |  |  |  |  |
| ***TDG*** | **rs3829301**^d^ |  |  |  |  |  |  |  |  |  |  |  |  |
|  |  | **Missing** | 0 | 1 |  |  |  |  |  |  |  |  |  |
|  |  | **AA** | 0 | 303 | 302.7 |  |  |  | 2.7 | 2.4 | 3.0 | - |  |
|  |  | **AC** | 1 | 29 | 29.6 |  |  |  | 2.9 | 2.0 | 4.3 | 7.4 |  |
|  |  | **CC** | 2 | 1 | 0.7 | 0.12 | *0.73* |  | 0.2 | 0.0 | 1.2 | -94.4 | *0.02* |
|  |  | **AC+CC** |  | 30 |  |  |  |  | 2.6 | 1.8 | 3.8 | -2.5 | *0.90* |
|  |  |  |  |  |  |  |  |  |  |  |  |  |  |
| ***TDG*** | **rs4135113** |  |  |  |  |  |  |  |  |  |  |  |  |
|  |  | **Missing** | 0 | 3 |  |  |  |  |  |  |  |  |  |
|  |  | **GG** | 0 | 314 | 314.2 |  |  |  | 2.7 | 2.4 | 3.0 | - |  |
|  |  | **AG** | 1 | 16 | 15.6 | 0.01 | *0.92* |  | 2.6 | 1.6 | 4.5 | -1.9 | *0.94* |
|  |  |  |  |  |  |  |  |  |  |  |  |  |  |
| ***TDG*** | **rs2629768**^d^ |  |  |  |  |  |  |  |  |  |  |  |  |
|  |  | **Missing** | 0 | 13 |  |  |  |  |  |  |  |  |  |
|  |  | **GG** | 0 | 138 | 138.4 |  |  |  | 2.5 | 2.2 | 2.9 | - |  |
|  |  | **GA** | 1 | 73 | 72.2 |  |  |  | 3.1 | 2.4 | 4.0 | 24.2 |  |
|  |  | **AA** | 2 | 9 | 9.4 | 0.03 | *0.87* |  | 2.5 | 1.3 | 5.0 | 0.6 | *0.31* |
|  |  | **GA+AA** |  | 82 |  |  |  |  | 3.0 | 2.4 | 3.8 | 21.3 | *0.15* |
|  |  |  |  |  |  |  |  |  |  |  |  |  |  |
| ***TDG*** | **rs4135064**^d^ |  |  |  |  |  |  |  |  |  |  |  |  |
|  |  | **Missing** | 0 | 1 |  |  |  |  |  |  |  |  |  |
|  |  | **CC** | 0 | 280 | 279.3 |  |  |  | 1.0 | 1.0 | 1.0 | - |  |
|  |  | **CT** | 1 | 49 | 50.4 |  |  |  | 1.0 | 1.0 | 1.0 | 0.0 |  |
|  |  | **TT** | 2 | 3 | 2.3 | 0.27 | *0.60* |  | 2.6 | 2.2 | 3.1 | 158.9 | *0.99* |
|  |  | **CT+TT** |  | 52 |  |  |  |  | 2.7 | 2.3 | 3.2 | 171.4 | *0.93* |
|  |  |  |  |  |  |  |  |  |  |  |  |  |  |
| ***TDG*** | **rs322107**^d^ |  |  |  |  |  |  |  |  |  |  |  |  |
|  |  | **Missing** | 0 | 5 |  |  |  |  |  |  |  |  |  |
|  |  | **CC** | 0 | 242 | 240.7 |  |  |  | 2.6 | 2.2 | 2.9 | - |  |
|  |  | **CT** | 1 | 78 | 80.5 |  |  |  | 2.9 | 2.3 | 3.7 | 14.2 |  |
|  |  | **TT** | 2 | 8 | 6.7 | 0.32 | *0.57* |  | 2.4 | 1.1 | 5.0 | -7.2 | *0.60* |
|  |  | **CT+TT** |  | 86 |  |  |  |  | 2.9 | 2.3 | 3.6 | 12.1 | *0.39* |
|  |  |  |  |  |  |  |  |  |  |  |  |  |  |
| ***TDG*** | **rs4135061** |  |  |  |  |  |  |  |  |  |  |  |  |
|  |  | **Missing** | 0 | 2 |  |  |  |  |  |  |  |  |  |
|  |  | **AA** | 0 | 185 | 182.8 |  |  |  | 2.6 | 2.2 | 3.0 | - |  |
|  |  | **AG** | 1 | 122 | 126.3 |  |  |  | 3.0 | 2.5 | 3.6 | 15.4 |  |
|  |  | **GG** | 2 | 24 | 21.8 | 0.39 | *0.53* |  | 2.1 | 1.4 | 3.2 | -19.7 | *0.24* |
|  |  |  |  |  |  |  |  |  |  |  |  |  |  |
| ***TDG*** | **rs4135081** |  |  |  |  |  |  |  |  |  |  |  |  |
|  |  | **Missing** | 1 | 1 |  |  |  |  |  |  |  |  |  |
|  |  | **AA** | 0 | 114 | 109.3 |  |  |  | 2.9 | 2.4 | 3.5 | - |  |
|  |  | **AG** | 1 | 153 | 162.4 |  |  |  | 2.6 | 2.2 | 3.1 | -9.9 |  |
|  |  | **GG** | 2 | 65 | 60.3 | 1.11 | *0.29* |  | 2.6 | 2.0 | 3.4 | -8.1 | *0.72* |
|  |  |  |  |  |  |  |  |  |  |  |  |  |  |
| ***TDG*** | **rs322109**^d^ |  |  |  |  |  |  |  |  |  |  |  |  |
|  |  | **Missing** | 0 | 18 |  |  |  |  |  |  |  |  |  |
|  |  | **AA** | 0 | 262 | 256.1 |  |  |  | 2.7 | 2.4 | 3.1 | - |  |
|  |  | **AG** | 1 | 44 | 55.9 |  |  |  | 2.2 | 1.6 | 3.0 | -19.2 |  |
|  |  | **GG** | 2 | 9 | 3.1 | 14.27 | *< 0.001* |  | 2.0 | 1.0 | 3.9 | -27.8 | *0.33* |
|  |  | **AG+GG** |  | 53 |  |  |  |  | 2.2 | 1.6 | 2.9 | -20.7 | *0.14* |
|  |  |  |  |  |  |  |  |  |  |  |  |  |  |
| ***TDG*** | **rs4135093** |  |  |  |  |  |  |  |  |  |  |  |  |
|  |  | **Missing** | 1 | 14 |  |  |  |  |  |  |  |  |  |
|  |  | **TT** | 0 | 111 | 109.6 |  |  |  | 2.7 | 2.2 | 3.3 | - |  |
|  |  | **TC** | 1 | 152 | 154.8 |  |  |  | 2.7 | 2.3 | 3.2 | 0.4 |  |
|  |  | **CC** | 2 | 56 | 54.6 | 0.10 | *0.75* |  | 2.6 | 2.0 | 3.5 | -2.1 | *0.99* |
|  |  |  |  |  |  |  |  |  |  |  |  |  |  |
| ***TDG*** | **rs4135094**^d^ |  |  |  |  |  |  |  |  |  |  |  |  |
|  |  | **Missing** | 0 | 4 |  |  |  |  |  |  |  |  |  |
|  |  | **TT** | 0 | 271 | 271.7 |  |  |  | 2.6 | 2.3 | 2.9 | - |  |
|  |  | **TC** | 1 | 56 | 54.5 |  |  |  | 3.3 | 2.5 | 4.4 | 28.5 |  |
|  |  | **CC** | 2 | 2 | 2.7 | 0.24 | *0.62* |  | 3.3 | 0.8 | 14.3 | 27.3 | *0.26* |
|  |  | **TC+CC** |  | 58 |  |  |  |  | 3.3 | 2.5 | 4.4 | 28.5 | *0.10* |
|  |  |  |  |  |  |  |  |  |  |  |  |  |  |
| ***TDG*** | **rs167715**^d^ |  |  |  |  |  |  |  |  |  |  |  |  |
|  |  | **Missing** | 0 | 1 |  |  |  |  |  |  |  |  |  |
|  |  | **TT** | 0 | 264 | 259.5 |  |  |  | 2.7 | 2.4 | 3.1 | - |  |
|  |  | **TC** | 1 | 59 | 68.1 |  |  |  | 2.6 | 2.0 | 3.5 | -2.9 |  |
|  |  | **CC** | 2 | 9 | 4.5 | 5.90 | *0.02* |  | 2.0 | 1.0 | 4.0 | -27.4 | *0.66* |
|  |  | **TC+CC** |  | 68 |  |  |  |  | 2.5 | 2.0 | 3.3 | -6.6 | *0.63* |
|  |  |  |  |  |  |  |  |  |  |  |  |  |  |
| ***UNG*** | **rs3219245**^d^ |  |  |  |  |  |  |  |  |  |  |  |  |
|  |  | **Missing** | 0 | 4 |  |  |  |  |  |  |  |  |  |
|  |  | **GG** | 0 | 275 | 272.6 |  |  |  | 2.8 | 2.5 | 3.2 | - |  |
|  |  | **GT** | 1 | 49 | 53.7 |  |  |  | 2.1 | 1.5 | 2.8 | -26.3 |  |
|  |  | **TT** | 2 | 5 | 2.6 | 2.53 | *0.11* |  | 2.2 | 0.9 | 5.6 | -21.6 | *0.16* |
|  |  | **GT+TT** |  | 54 |  |  |  |  | 2.1 | 1.6 | 2.8 | -25.9 | *0.06* |
|  |  |  |  |  |  |  |  |  |  |  |  |  |  |
| ***UNG*** | **rs246079** |  |  |  |  |  |  |  |  |  |  |  |  |
|  |  | **Missing** | 1 | 2 |  |  |  |  |  |  |  |  |  |
|  |  | **AA** | 0 | 115 | 110.2 |  |  |  | 2.8 | 2.3 | 3.4 | - |  |
|  |  | **AG** | 1 | 152 | 161.6 |  |  |  | 2.8 | 2.3 | 3.3 | -2.1 |  |
|  |  | **GG** | 2 | 64 | 59.2 | 1.16 | *0.28* |  | 2.4 | 1.9 | 3.1 | -14.1 | *0.63* |
|  |  |  |  |  |  |  |  |  |  |  |  |  |  |
| ***XRCC1*** | **rs939461**^d^ |  |  |  |  |  |  |  |  |  |  |  |  |
|  |  | **Missing** | 0 | 2 |  |  |  |  |  |  |  |  |  |
|  |  | **AA** | 0 | 274 | 273.7 |  |  |  | 2.7 | 2.4 | 3.1 | - |  |
|  |  | **AC** | 1 | 54 | 54.6 |  |  |  | 2.6 | 2.0 | 3.5 | -4.2 |  |
|  |  | **CC** | 2 | 3 | 2.7 | 0.04 | *0.85* |  | 2.3 | 0.7 | 7.8 | -14.4 | *0.94* |
|  |  | **AC+CC** |  | 57 |  |  |  |  | 2.6 | 2.0 | 3.4 | -4.7 | *0.75* |
|  |  |  |  |  |  |  |  |  |  |  |  |  |  |
| ***XRCC1*** | **rs3213247 ^c^** |  |  |  |  |  |  |  |  |  |  |  |  |
|  |  | **Missing** | 0 | 2 |  |  |  |  |  |  |  |  |  |
|  |  | **GG** | 0 | 288 | 288.5 |  |  |  | 2.7 | 2.4 | 3.1 | - |  |
|  |  | **GT** | 1 | 42 | 41.1 |  |  |  | 2.5 | 1.8 | 3.4 | -8.7 |  |
|  |  | **TT** | 2 | 1 | 1.5 | 0.17 | *0.68* |  | 6.3 | 0.8 | 52.3 | 131.4 | *0.64* |
|  |  | **GT+TT** |  | 43 |  |  |  |  | 2.5 | 1.9 | 3.5 | -6.8 | *0.69* |
|  |  |  |  |  |  |  |  |  |  |  |  |  |  |
| ***XRCC1*** | **rs939460** |  |  |  |  |  |  |  |  |  |  |  |  |
|  |  | **Missing** | 0 | 7 |  |  |  |  |  |  |  |  |  |
|  |  | **GG** | 0 | 221 | 213.8 |  |  |  | 2.8 | 2.4 | 3.2 | - |  |
|  |  | **GA** | 1 | 86 | 100.4 |  |  |  | 2.7 | 2.2 | 3.4 | -2.3 |  |
|  |  | **AA** | 2 | 19 | 11.8 | 6.72 | *0.01* |  | 2.3 | 1.5 | 3.8 | -16.0 | *0.78* |
|  |  |  |  |  |  |  |  |  |  |  |  |  |  |
| ***XRCC1*** | **rs25487** |  |  |  |  |  |  |  |  |  |  |  |  |
|  |  | **Missing** | 1 | 1 |  |  |  |  |  |  |  |  |  |
|  |  | **GG** | 0 | 123 | 126.0 |  |  |  | 2.7 | 2.3 | 3.3 | - |  |
|  |  | **GA** | 1 | 163 | 157.1 |  |  |  | 2.6 | 2.2 | 3.1 | -4.1 |  |
|  |  | **AA** | 2 | 46 | 49.0 | 0.47 | *0.49* |  | 2.8 | 2.1 | 3.9 | 3.8 | *0.89* |
|  |  |  |  |  |  |  |  |  |  |  |  |  |  |
| ***XRCC1*** | **rs25489**^d^ |  |  |  |  |  |  |  |  |  |  |  |  |
|  |  | **Missing** | 0 | 0 |  |  |  |  |  |  |  |  |  |
|  |  | **GG** | 0 | 305 | 303.7 |  |  |  | 2.7 | 2.4 | 3.0 | - |  |
|  |  | **GA** | 1 | 26 | 28.6 |  |  |  | 3.1 | 2.1 | 4.7 | 17.8 |  |
|  |  | **AA** | 2 | 2 | 0.7 | 2.85 | *0.09* |  | 4.5 | 1.0 | 19.7 | 70.2 | *0.59* |
|  |  | **GA+AA** |  | 28 |  |  |  |  | 3.2 | 2.2 | 4.7 | 20.9 | *0.36* |
|  |  |  |  |  |  |  |  |  |  |  |  |  |  |
| ***XRCC1*** | **rs1001581** |  |  |  |  |  |  |  |  |  |  |  |  |
|  |  | **Missing** | 1 | 7 |  |  |  |  |  |  |  |  |  |
|  |  | **CC** | 0 | 114 | 117.8 |  |  |  | 2.8 | 2.3 | 3.4 | - |  |
|  |  | **CT** | 1 | 164 | 156.3 |  |  |  | 2.6 | 2.2 | 3.0 | -8.3 |  |
|  |  | **TT** | 2 | 48 | 51.8 | 0.79 | *0.37* |  | 2.8 | 2.1 | 3.8 | 1.2 | *0.74* |
|  |  |  |  |  |  |  |  |  |  |  |  |  |  |
| ***XRCC1*** | **rs2307191** |  |  |  |  |  |  |  |  |  |  |  |  |
|  |  | **Missing** | 0 | 8 |  |  |  |  |  |  |  |  |  |
|  |  | **CC** | 0 | 325 |  |  |  |  | 2.7 | 2.4 | 3.0 | - | *NA* |
|  |  |  |  |  |  |  |  |  |  |  |  |  |  |
| ***XRCC1*** | **rs3213403**^d^ |  |  |  |  |  |  |  |  |  |  |  |  |
|  |  | **Missing** | 0 | 1 |  |  |  |  |  |  |  |  |  |
|  |  | **AA** | 0 | 291 | 290.4 |  |  |  | 2.6 | 2.3 | 2.9 | - |  |
|  |  | **AG** | 1 | 39 | 40.2 |  |  |  | 3.5 | 2.5 | 4.8 | 32.8 |  |
|  |  | **GG** | 2 | 2 | 1.4 | 0.30 | *0.58* |  | 2.1 | 0.5 | 9.3 | -18.6 | *0.28* |
|  |  | **AG+GG** |  | 41 |  |  |  |  | 3.4 | 2.4 | 4.7 | 29.7 | *0.14* |
|  |  |  |  |  |  |  |  |  |  |  |  |  |  |
| ***XRCC1*** | **rs915927** |  |  |  |  |  |  |  |  |  |  |  |  |
|  |  | **Missing** | 1 | 13 |  |  |  |  |  |  |  |  |  |
|  |  | **AA** | 0 | 106 | 107.5 |  |  |  | 3.1 | 2.5 | 3.8 | - |  |
|  |  | **AG** | 1 | 159 | 155.9 |  |  |  | 2.6 | 2.2 | 3.0 | -16.9 |  |
|  |  | **GG** | 2 | 55 | 56.5 | 0.12 | *0.73* |  | 2.4 | 1.8 | 3.2 | -21.6 | *0.25* |
|  |  |  |  |  |  |  |  |  |  |  |  |  |  |
| ***XRCC1*** | **rs3213255** |  |  |  |  |  |  |  |  |  |  |  |  |
|  |  | **Missing** | 1 | 15 |  |  |  |  |  |  |  |  |  |
|  |  | **TT** | 0 | 122 | 120.8 |  |  |  | 3.1 | 2.5 | 3.7 | - |  |
|  |  | **TC** | 1 | 148 | 150.4 |  |  |  | 2.6 | 2.2 | 3.1 | -13.6 |  |
|  |  | **CC** | 2 | 48 | 46.8 | 0.08 | *0.78* |  | 2.4 | 1.8 | 3.2 | -22.8 | *0.29* |
|  |  |  |  |  |  |  |  |  |  |  |  |  |  |
| ***XRCC1*** | **rs25496** |  |  |  |  |  |  |  |  |  |  |  |  |
|  |  | **Missing** | 0 | 4 |  |  |  |  |  |  |  |  |  |
|  |  | **TT** | 0 | 329 |  |  |  |  | 2.7 | 2.4 | 3.0 | - | *NA* |

Abbreviations: CL, confidence limit; Diff., difference; HWE, Hardy-Weinberg Equilibrium; LL, lower limit; MAP, Markers of Adenomatous Polyps; Prop. diff., proportional difference; SNP, single nucleotide polymorphism; UL, upper limit

^a^ Geometric means, 95% confidence limits, and *P*-values from general linear models, adjusted for sex (male/female) and body mass index (continuous)

^b^ Based on minor allele frequency in the European population in the 1000 Genomes Project Phase 3 (ensembl.org OR ncbi.nlm.nih.gov/projects/SNP)

^c^ Proportional difference, in percent, between mean plasma high sensitivity C-reactive protein concentration among those with a variant genotype relative to those with the common homozygous genotype; i.e.: ([variant mean - common mean] / common mean) x 100%

^d^ Heterozygous and/or variant homozygous genotypes with ≤ 10 participants were combined

**Supplemental Table 6.** Antioxidant enzyme genetic risk score genes, their antioxidant functions, and SNPs included

| **Gene** | **Functions** | **SNP rs ID** | **Consequence** |
| --- | --- | --- | --- |
| *CAT* | Defends against superoxide and hydrogen peroxide; constitutes a primary defense against oxidative stress (Röhrdanz and Kahl, 1998) | rs16925614 | Intron Variant |
| *MnSoD* | Prevents disruption of mitochondrial membrane potential; catalyzes dismutation of superoxide radicals (Röhrdanz and Kahl, 1998; Mäntymaa et al., 2000) | rs4880 | Missense Variant |

Abbreviations: ID, identifier; SNP, single nucleotide polymorphism

^a^ Inclusion criteria: Genes and SNPs from Supplemental Table 1 included in the AE GRS if in Supplemental Table 6 the proportional mean differences in plasma hsCRP concentrations were > 5% plus p ≤ 0.05, or the proportional mean differences in hsCRP concentrations were > 10% plus p ≤ 0.15

**Supplemental Table 7.** DNA base excision repair genetic risk score genes, their functions, and SNPs included

| **Gene** | **Functions** | **SNP rs ID** | **Consequence** |
| --- | --- | --- | --- |
| *MUTYH* | Contributes to oxidative damage repair by removing mismatched 8-oxoG adenine (Sampson et al., 2005; Nielsen et al., 2011) | rs3219484 | Missense Variant |
|  |  |  |  |
| *SMUG1* | Repairs DNA damage by removing uracil from single- and double-stranded DNA in nuclear chromatin (Nilsen et al., 2001; Broderick et al., 2006) | rs971 | Intron Variant |
|  |  |  |  |
| *TDG* | Involved in DNA demethylation; repairs G/T and G/U mismatches via removing thymine and uracil moieties (He et al., 2011; Wu and Zhang, 2017) | rs2629768 | Intron Variant |
|  |  | rs322109 | Intron Variant |
|  |  | rs4135094 | Intron Variant |
|  |  |  |  |
| *UNG* | Repairs mutagenic G/U mismatches caused by deamination of cytosine (Krokan et al., 2001) | rs3219245 | Intron Variant |
|  |  |  |  |
| *XRCC1* | Repairs single-stranded DNA breaks (Duell et al., 2000; Thompson and West, 2000) | rs3213403 | None |

Abbreviations: ID, identifier; SNP, single nucleotide polymorphism; 8-oxoG, 8-dihydro-2′ -deoxyguanosine

^a^ Inclusion criteria: Genes and SNPs from Supplemental Table 2 included in the BER GRS if in Supplemental Table 7 the proportional mean differences in hsCRP concentrations were > 10% plus p ≤ 0.15

**Supplemental Table 8.** Mean^a^ plasma high sensitivity C-reactive protein concentrations, according to tertiles of dietary and lifestyle inflammation scores^b^, in the pooled MAP I and MAP II cross-sectional studies (n = 333)

| **Model/scores, tertiles** | **Scores' tertile medians** | **Plasma hsCRP, µg/mL** | | | | |
| --- | --- | --- | --- | --- | --- | --- |
|  |  | n^c^ | Means | 95% CI | Prop. diff.^d^ (%) | *P*-values |
|  |  |  |  |  |  |  |
| Crude^e^ |  |  |  |  |  |  |
| DIS tertiles |  |  |  |  |  |  |
| 1 | -1.60 | 111 | 2.3 | (1.9, 2.9) | Ref. |  |
| 2 | 1.07 | 111 | 2.7 | (2.2, 3.3) | 17.4 |  |
| 3 | 3.64 | 111 | 3.3 | (2.7, 4.0) | 43.5 | 0.07 |
| LIS tertiles |  |  |  |  |  |  |
| 1 | -0.34 | 116 | 1.9 | (1.6, 2.3) | Ref. |  |
| 2 | 0.50 | 106 | 2.5 | (2.0, 3.0) | 31.6 |  |
| 3 | 1.16 | 111 | 4.4 | (3.6, 5.4) | 131.6 | <0.001 |
| Multivariable-adjusted^f^ | |  |  |  |  |  |
| DIS tertiles |  |  |  |  |  |  |
| 1 | -1.60 | 111 | 2.7 | (2.0, 3.6) | Ref. |  |
| 2 | 1.07 | 111 | 2.8 | (2.1, 3.7) | 3.7 |  |
| 3 | 3.64 | 111 | 3.2 | (2.4, 4.2) | 18.5 | 0.66 |
| LIS tertiles |  |  |  |  |  |  |
| 1 | -0.34 | 116 | 2.0 | (1.6, 2.5) | Ref. |  |
| 2 | 0.50 | 106 | 2.7 | (2.1, 3.4) | 35.0 |  |
| 3 | 1.16 | 111 | 4.4 | (3.5, 5.5) | 120.0 | <0.001 |

Abbreviations: CI, confidence interval; DIS, dietary inflammation scores; hsCRP, high sensitivity C-reactive protein; MAP, Markers of Adenomatous Polyps; LIS, lifestyle inflammation scores; Prop. diff., proportional difference

^a^ Geometric means and 95% confidence intervals and *P*-values from general linear models

^b^ For calculation of the scores, see text; a higher score represents more inflammatory relative to anti-inflammatory exposures

^c^ Differences in numbers of participants for the two biomarkers due to availability of serum samples for the two biomarker assays

^d^ Proportional difference calculated as (comparison group mean - reference group mean) / (reference group mean) x 100%

^e^ No covariates in the model

^f^ Covariates in the DIS models: total energy intake, sex, education (less than high school, high school degree, college graduate or higher), current hormone replacement therapy use (among women), current smoking (yes/no), body mass index category, alcohol intake (none, moderate, heavy), physical activity level, study, and regular aspirin and/or other nonsteroidal anti-inflammatory drug use (≥ 1/wk or < 1/wk). Covariates in the LIS models: sex, education (less than high school, high school degree, college graduate or higher), current hormone replacement therapy use (among women), regular aspirin and/or other nonsteroidal anti-inflammatory drug use (≥ 1/wk or < 1/wk), and the DIS (continuous)

**Supplemental References**

Alam, M.A., Subhan, N., Rahman, M.M., Uddin, S.J., Reza, H.M., and Sarker, S.D. (2014). Effect of citrus flavonoids, naringin and naringenin, on metabolic syndrome and their mechanisms of action. *Adv Nutr* 5(4)**,** 404-417. doi: 10.3945/an.113.005603.

Böhm, F., Settergren, M., and Pernow, J. (2007). Vitamin C blocks vascular dysfunction and release of interleukin-6 induced by endothelin-1 in humans in vivo. *Atherosclerosis* 190(2)**,** 408-415. doi: 10.1016/j.atherosclerosis.2006.02.018.

Broderick, P., Bagratuni, T., Vijayakrishnan, J., Lubbe, S., Chandler, I., and Houlston, R.S. (2006). Evaluation of NTHL1, NEIL1, NEIL2, MPG, TDG, UNG and SMUG1 genes in familial colorectal cancer predisposition. *BMC Cancer* 6**,** 243. doi: 10.1186/1471-2407-6-243.

Brown, A.A., and Hu, F.B. (2001). Dietary modulation of endothelial function: implications for cardiovascular disease. *Am J Clin Nutr* 73(4)**,** 673-686. doi: 10.1093/ajcn/73.4.673.

Burton-Freeman, B., and Sesso, H.D. (2014). Whole food versus supplement: comparing the clinical evidence of tomato intake and lycopene supplementation on cardiovascular risk factors. *Adv Nutr* 5(5)**,** 457-485. doi: 10.3945/an.114.005231.

Calder, P.C. (2010). Omega-3 fatty acids and inflammatory processes. *Nutrients* 2(3)**,** 355-374. doi: 10.3390/nu2030355.

Calder, P.C., Ahluwalia, N., Brouns, F., Buetler, T., Clement, K., Cunningham, K., et al. (2011). Dietary factors and low-grade inflammation in relation to overweight and obesity. *Br J Nutr* 106 Suppl 3**,** S5-78. doi: 10.1017/s0007114511005460.

Casas-Agustench, P., Bulló, M., and Salas-Salvadó, J. (2010). Nuts, inflammation and insulin resistance. *Asia Pac J Clin Nutr* 19(1)**,** 124-130.

Chassaing, B., Koren, O., Goodrich, J.K., Poole, A.C., Srinivasan, S., Ley, R.E., et al. (2015). Dietary emulsifiers impact the mouse gut microbiota promoting colitis and metabolic syndrome. *Nature* 519(7541)**,** 92-96. doi: 10.1038/nature14232.

Codoñer-Franch, P., Betoret, E., Betoret, N., López-Jaén, A.B., Valls-Bellés, V., and Fito, P. (2013). Dried apples enriched with mandarin juice by vacuum impregnation improve antioxidant capacity and decrease inflammation in obese children. *Nutr Hosp* 28(4)**,** 1177-1183. doi: 10.3305/nh.2013.28.4.6580.

Das, S.K., and Vasudevan, D.M. (2007). Alcohol-induced oxidative stress. *Life Sci* 81(3)**,** 177-187. doi: 10.1016/j.lfs.2007.05.005.

Dash, C., Goodman, M., Flanders, W.D., Mink, P.J., McCullough, M.L., and Bostick, R.M. (2013). Using pathway-specific comprehensive exposure scores in epidemiology: application to oxidative balance in a pooled case-control study of incident, sporadic colorectal adenomas. *Am J Epidemiol* 178(4)**,** 610-624. doi: 10.1093/aje/kwt007.

Dower, J.I., Geleijnse, J.M., Gijsbers, L., Schalkwijk, C., Kromhout, D., and Hollman, P.C. (2015). Supplementation of the Pure Flavonoids Epicatechin and Quercetin Affects Some Biomarkers of Endothelial Dysfunction and Inflammation in (Pre)Hypertensive Adults: A Randomized Double-Blind, Placebo-Controlled, Crossover Trial. *J Nutr* 145(7)**,** 1459-1463. doi: 10.3945/jn.115.211888.

Du, S.Y., Zhang, Y.L., Bai, R.X., Ai, Z.L., Xie, B.S., and Yang, H.Y. (2015). Lutein prevents alcohol-induced liver disease in rats by modulating oxidative stress and inflammation. *Int J Clin Exp Med* 8(6)**,** 8785-8793.

Duell, E.J., Wiencke, J.K., Cheng, T.J., Varkonyi, A., Zuo, Z.F., Ashok, T.D., et al. (2000). Polymorphisms in the DNA repair genes XRCC1 and ERCC2 and biomarkers of DNA damage in human blood mononuclear cells. *Carcinogenesis* 21(5)**,** 965-971. doi: 10.1093/carcin/21.5.965.

Espley, R.V., Butts, C.A., Laing, W.A., Martell, S., Smith, H., McGhie, T.K., et al. (2014). Dietary flavonoids from modified apple reduce inflammation markers and modulate gut microbiota in mice. *J Nutr* 144(2)**,** 146-154. doi: 10.3945/jn.113.182659.

Fortis-Barrera, Á., Alarcón-Aguilar, F.J., Banderas-Dorantes, T., Díaz-Flores, M., Román-Ramos, R., Cruz, M., et al. (2013). Cucurbita ficifolia Bouché (Cucurbitaceae) and D-chiro-inositol modulate the redox state and inflammation in 3T3-L1 adipocytes. *J Pharm Pharmacol* 65(10)**,** 1563-1576. doi: 10.1111/jphp.12119.

Furukawa, S., Fujita, T., Shimabukuro, M., Iwaki, M., Yamada, Y., Nakajima, Y., et al. (2004). Increased oxidative stress in obesity and its impact on metabolic syndrome. *J Clin Invest* 114(12)**,** 1752-1761. doi: 10.1172/jci21625.

Ghanim, H., Mohanty, P., Pathak, R., Chaudhuri, A., Sia, C.L., and Dandona, P. (2007). Orange juice or fructose intake does not induce oxidative and inflammatory response. *Diabetes Care* 30(6)**,** 1406-1411. doi: 10.2337/dc06-1458.

Giugliano, D., Ceriello, A., and Esposito, K. (2006). The effects of diet on inflammation: emphasis on the metabolic syndrome. *J Am Coll Cardiol* 48(4)**,** 677-685. doi: 10.1016/j.jacc.2006.03.052.

Gomez-Cabrera, M.C., Domenech, E., and Viña, J. (2008). Moderate exercise is an antioxidant: upregulation of antioxidant genes by training. *Free Radic Biol Med* 44(2)**,** 126-131. doi: 10.1016/j.freeradbiomed.2007.02.001.

Govers, M.J., Termont, D.S., Lapré, J.A., Kleibeuker, J.H., Vonk, R.J., and Van der Meer, R. (1996). Calcium in milk products precipitates intestinal fatty acids and secondary bile acids and thus inhibits colonic cytotoxicity in humans. *Cancer Res* 56(14)**,** 3270-3275.

Guardia, T., Rotelli, A.E., Juarez, A.O., and Pelzer, L.E. (2001). Anti-inflammatory properties of plant flavonoids. Effects of rutin, quercetin and hesperidin on adjuvant arthritis in rat. *Farmaco* 56(9)**,** 683-687. doi: 10.1016/s0014-827x(01)01111-9.

Guzik, T.J., Korbut, R., and Adamek-Guzik, T. (2003). Nitric oxide and superoxide in inflammation and immune regulation. *J Physiol Pharmacol* 54(4)**,** 469-487.

Hale, L.P., Chichlowski, M., Trinh, C.T., and Greer, P.K. (2010). Dietary supplementation with fresh pineapple juice decreases inflammation and colonic neoplasia in IL-10-deficient mice with colitis. *Inflamm Bowel Dis* 16(12)**,** 2012-2021. doi: 10.1002/ibd.21320.

Hartman, T.J., Albert, P.S., Zhang, Z., Bagshaw, D., Kris-Etherton, P.M., Ulbrecht, J., et al. (2010). Consumption of a legume-enriched, low-glycemic index diet is associated with biomarkers of insulin resistance and inflammation among men at risk for colorectal cancer. *J Nutr* 140(1)**,** 60-67. doi: 10.3945/jn.109.114249.

He, Y.F., Li, B.Z., Li, Z., Liu, P., Wang, Y., Tang, Q., et al. (2011). Tet-mediated formation of 5-carboxylcytosine and its excision by TDG in mammalian DNA. *Science* 333(6047)**,** 1303-1307. doi: 10.1126/science.1210944.

Hussain, T., Tan, B., Liu, G., Murtaza, G., Rahu, N., Saleem, M., et al. (2017). Modulatory Mechanism of Polyphenols and Nrf2 Signaling Pathway in LPS Challenged Pregnancy Disorders. *Oxid Med Cell Longev* 2017**,** 8254289. doi: 10.1155/2017/8254289.

Jacob, K., Periago, M.J., Böhm, V., and Berruezo, G.R. (2008). Influence of lycopene and vitamin C from tomato juice on biomarkers of oxidative stress and inflammation. *Br J Nutr* 99(1)**,** 137-146. doi: 10.1017/s0007114507791894.

Jia, Q., Cheng, W., Yue, Y., Hu, Y., Zhang, J., Pan, X., et al. (2015). Cucurbitacin E inhibits TNF-α-induced inflammatory cytokine production in human synoviocyte MH7A cells via suppression of PI3K/Akt/NF-κB pathways. *Int Immunopharmacol* 29(2)**,** 884-890. doi: 10.1016/j.intimp.2015.08.026.

Johnson, E.J. (2014). Role of lutein and zeaxanthin in visual and cognitive function throughout the lifespan. *Nutr Rev* 72(9)**,** 605-612. doi: 10.1111/nure.12133.

Johnson, M., Pace, R.D., and McElhenney, W.H. (2018). Green leafy vegetables in diets with a 25:1 omega-6/omega-3 fatty acid ratio modify the erythrocyte fatty acid profile of spontaneously hypertensive rats. *Lipids Health Dis* 17(1)**,** 140. doi: 10.1186/s12944-018-0723-7.

Kallio, P., Kolehmainen, M., Laaksonen, D.E., Kekäläinen, J., Salopuro, T., Sivenius, K., et al. (2007). Dietary carbohydrate modification induces alterations in gene expression in abdominal subcutaneous adipose tissue in persons with the metabolic syndrome: the FUNGENUT Study. *Am J Clin Nutr* 85(5)**,** 1417-1427. doi: 10.1093/ajcn/85.5.1417.

Kelly, K.B., Kennelly, J.P., Ordonez, M., Nelson, R., Leonard, K., Stabler, S., et al. (2016). Excess Folic Acid Increases Lipid Storage, Weight Gain, and Adipose Tissue Inflammation in High Fat Diet-Fed Rats. *Nutrients* 8(10). doi: 10.3390/nu8100594.

Knekt, P., Kumpulainen, J., Järvinen, R., Rissanen, H., Heliövaara, M., Reunanen, A., et al. (2002). Flavonoid intake and risk of chronic diseases. *Am J Clin Nutr* 76(3)**,** 560-568. doi: 10.1093/ajcn/76.3.560.

Krokan, H.E., Otterlei, M., Nilsen, H., Kavli, B., Skorpen, F., Andersen, S., et al. (2001). Properties and functions of human uracil-DNA glycosylase from the UNG gene. *Prog Nucleic Acid Res Mol Biol* 68**,** 365-386.

Ludwig, D.S. (2002). The glycemic index: physiological mechanisms relating to obesity, diabetes, and cardiovascular disease. *Jama* 287(18)**,** 2414-2423. doi: 10.1001/jama.287.18.2414.

Mäntymaa, P., Siitonen, T., Guttorm, T., Säily, M., Kinnula, V., Savolainen, E.R., et al. (2000). Induction of mitochondrial manganese superoxide dismutase confers resistance to apoptosis in acute myeloblastic leukaemia cells exposed to etoposide. *Br J Haematol* 108(3)**,** 574-581. doi: 10.1046/j.1365-2141.2000.01852.x.

Markovits, N., Ben Amotz, A., and Levy, Y. (2009). The effect of tomato-derived lycopene on low carotenoids and enhanced systemic inflammation and oxidation in severe obesity. *Isr Med Assoc J* 11(10)**,** 598-601.

Mathews, M.J., Liebenberg, L., and Mathews, E.H. (2015). The mechanism by which moderate alcohol consumption influences coronary heart disease. *Nutr J* 14**,** 33. doi: 10.1186/s12937-015-0011-6.

McCarty, M.F. (1999). Interleukin-6 as a central mediator of cardiovascular risk associated with chronic inflammation, smoking, diabetes, and visceral obesity: down-regulation with essential fatty acids, ethanol and pentoxifylline. *Med Hypotheses* 52(5)**,** 465-477. doi: 10.1054/mehy.1997.0684.

Nidhi, B., Sharavana, G., Ramaprasad, T.R., and Vallikannan, B. (2015). Lutein derived fragments exhibit higher antioxidant and anti-inflammatory properties than lutein in lipopolysaccharide induced inflammation in rats. *Food Funct* 6(2)**,** 450-460. doi: 10.1039/c4fo00606b.

Nielsen, M., Morreau, H., Vasen, H.F., and Hes, F.J. (2011). MUTYH-associated polyposis (MAP). *Crit Rev Oncol Hematol* 79(1)**,** 1-16. doi: 10.1016/j.critrevonc.2010.05.011.

Nilsen, H., Haushalter, K.A., Robins, P., Barnes, D.E., Verdine, G.L., and Lindahl, T. (2001). Excision of deaminated cytosine from the vertebrate genome: role of the SMUG1 uracil–DNA glycosylase. *EMBO J* 20(15)**,** 4278-4286.

Obeid, R., Kirsch, S.H., Kasoha, M., Eckert, R., and Herrmann, W. (2011). Concentrations of unmetabolized folic acid and primary folate forms in plasma after folic acid treatment in older adults. *Metabolism* 60(5)**,** 673-680. doi: 10.1016/j.metabol.2010.06.020.

Park, J.B. (2018). Javamide-II Found in Coffee Is Better than Caffeine at Suppressing TNF-α Production in PMA/PHA-Treated Lymphocytic Jurkat Cells. *J Agric Food Chem* 66(26)**,** 6782-6789. doi: 10.1021/acs.jafc.8b01885.

Prior, R.L., Gu, L., Wu, X., Jacob, R.A., Sotoudeh, G., Kader, A.A., et al. (2007). Plasma antioxidant capacity changes following a meal as a measure of the ability of a food to alter in vivo antioxidant status. *J Am Coll Nutr* 26(2)**,** 170-181. doi: 10.1080/07315724.2007.10719599.

Rao, A.V. (2002). Lycopene, tomatoes, and the prevention of coronary heart disease. *Exp Biol Med (Maywood)* 227(10)**,** 908-913. doi: 10.1177/153537020222701011.

Röhrdanz, E., and Kahl, R. (1998). Alterations of antioxidant enzyme expression in response to hydrogen peroxide. *Free Radic Biol Med* 24(1)**,** 27-38. doi: 10.1016/s0891-5849(97)00159-7.

Sampson, J.R., Jones, S., Dolwani, S., and Cheadle, J.P. (2005). MutYH (MYH) and colorectal cancer. *Biochem Soc Trans* 33(Pt 4)**,** 679-683. doi: 10.1042/bst0330679.

Sharma, D., Rawat, I., and Goel, H.C. (2015). Anticancer and anti-inflammatory activities of some dietary cucurbits. *Indian J Exp Biol* 53(4)**,** 216-221.

Simopoulos, A.P. (2002). Omega-3 fatty acids in inflammation and autoimmune diseases. *J Am Coll Nutr* 21(6)**,** 495-505. doi: 10.1080/07315724.2002.10719248.

Sommerburg, O., Keunen, J.E., Bird, A.C., and van Kuijk, F.J. (1998). Fruits and vegetables that are sources for lutein and zeaxanthin: the macular pigment in human eyes. *Br J Ophthalmol* 82(8)**,** 907-910. doi: 10.1136/bjo.82.8.907.

Thompson, L.H., and West, M.G. (2000). XRCC1 keeps DNA from getting stranded. *Mutat Res* 459(1)**,** 1-18. doi: 10.1016/s0921-8777(99)00058-0.

van Bussel, B.C., Henry, R.M., Ferreira, I., van Greevenbroek, M.M., van der Kallen, C.J., Twisk, J.W., et al. (2015). A healthy diet is associated with less endothelial dysfunction and less low-grade inflammation over a 7-year period in adults at risk of cardiovascular disease. *J Nutr* 145(3)**,** 532-540. doi: 10.3945/jn.114.201236.

van der Vaart, H., Postma, D.S., Timens, W., and ten Hacken, N.H. (2004). Acute effects of cigarette smoke on inflammation and oxidative stress: a review. *Thorax* 59(8)**,** 713-721. doi: 10.1136/thx.2003.012468.

van Woudenbergh, G.J., Kuijsten, A., Tigcheler, B., Sijbrands, E.J., van Rooij, F.J., Hofman, A., et al. (2012). Meat consumption and its association with C-reactive protein and incident type 2 diabetes: the Rotterdam Study. *Diabetes Care* 35(7)**,** 1499-1505. doi: 10.2337/dc11-1899.

Wall, R., Ross, R.P., Fitzgerald, G.F., and Stanton, C. (2010). Fatty acids from fish: the anti-inflammatory potential of long-chain omega-3 fatty acids. *Nutr Rev* 68(5)**,** 280-289. doi: 10.1111/j.1753-4887.2010.00287.x.

Wang, M.X., Jiao, J.H., Li, Z.Y., Liu, R.R., Shi, Q., and Ma, L. (2013). Lutein supplementation reduces plasma lipid peroxidation and C-reactive protein in healthy nonsmokers. *Atherosclerosis* 227(2)**,** 380-385. doi: 10.1016/j.atherosclerosis.2013.01.021.

White, D.L., and Collinson, A. (2013). Red meat, dietary heme iron, and risk of type 2 diabetes: the involvement of advanced lipoxidation endproducts. *Adv Nutr* 4(4)**,** 403-411. doi: 10.3945/an.113.003681.

Wu, D., Zhai, Q., and Shi, X. (2006). Alcohol-induced oxidative stress and cell responses. *J Gastroenterol Hepatol* 21 Suppl 3**,** S26-29. doi: 10.1111/j.1440-1746.2006.04589.x.

Wu, X., and Zhang, Y. (2017). TET-mediated active DNA demethylation: mechanism, function and beyond. *Nat Rev Genet* 18(9)**,** 517-534. doi: 10.1038/nrg.2017.33.

Zitvogel, L., Pietrocola, F., and Kroemer, G. (2017). Nutrition, inflammation and cancer. *Nat Immunol* 18(8)**,** 843-850. doi: 10.1038/ni.3754.
